# Systematic review of measurement properties of methods for objectively assessing masticatory performance

**DOI:** 10.1002/cre2.154

**Published:** 2019-01-31

**Authors:** Per Elgestad Stjernfeldt, Petteri Sjögren, Inger Wårdh, Anne‐Marie Boström

**Affiliations:** ^1^ Academic Centre for Geriatric Dentistry; Public dental care in Stockholm County Karolinska Institutet Sweden; ^2^ Oral Care AB Sweden; ^3^ Oral Diagnostics and Surgery unit, Dept. of Dental Medicine Karolinska Institutet Sweden; ^4^ Department of Neurobiology, Care Sciences and Society; Divison of nursing Karolinska Institutet, Huddinge, Sweden Haugesund Norway; ^5^ Karolinska University Hospital, Theme Aging Stockholm Sweden; ^6^ Stockholms Sjukhem R&D Unit Stockholm Sweden; ^7^ Western Norway University of Applied Sciences Bergen Norway

**Keywords:** COSMIN, masticatory performance, measurement error, reliability, responsiveness, validity

## Abstract

The objectives of this study is to identify methods for objectively assessing masticatory performance (MP) and to evaluate their measurement properties. A secondary objective was to identify any reported adverse events associated with the methods to assess MP. Bibliographic databases were searched, including MEDLINE, Embase, Web of Science Core Collection, Cochrane, and Cinahl databases. Eligible papers that satisfied predefined inclusion and exclusion criteria were appraised independently by two investigators. Four other investigators independently appraised any measurement properties of the assessment method according to the consensus‐based standards for the selection of health measurement instruments checklist. The qualities of the measurement properties were evaluated using predefined criteria. The level of evidence was rated by using data synthesis for each MP assessment method, where the rating was a product of methodological quality and measurement properties quality. All studies were quality assessed separately, initially, and subsequently for each method. Studies that described the use of identical assessment method received an individual score, and the pooled sum score resulted in an overall evidence synthesis. The level of evidence was synthesized across studies with an overall conclusion, that is, unknown, conflicting, limited, moderate, or strong evidence. Forty‐six out of 9,908 articles were appraised, and the assessment methods were categorized as comminution (*n* = 21), mixing ability (*n* = 23), or other methods (*n* = 2). Different measurement properties were identified, in decreasing order construct validity (*n* = 30), reliability (*n* = 22), measurement error (*n* = 9), criterion validity (*n* = 6), and responsiveness (*n* = 4). No adverse events associated with any assessment methods were reported. In a clinical setting or as a diagnostic method, there are no gold standard methods for assessing MP with a strong level of evidence for all measurement properties. All available assessment methods with variable level of evidence require lab‐intensive equipment, such as sieves or digital image software. Clinical trials with sufficient sample size, to infer trueness and precision, are needed for evaluating diagnostic values of available methods for assessing masticatory performance.

## 1 INTRODUCTION

A primary goal of dental treatment is to restore dental and oral function, including ability to masticate food. Masticatory performance is defined as ability to comminute or mix test food (van der Bilt, 2011) The most common method for assessing masticatory performance is a comminution method using a sieve. Test food is masticated, and then, food particles are separated using sieves with varying aperture sizes; the smaller the particles size, the better the masticatory performance. Dahlberg and Manley were among the first to introduce the sieve method (Dahlberg, 1942; Manly & Braley, 1950). They used test foods, such as peanuts and carrots, and later, silicone‐based materials were introduced.

Many years later, alternatives to the sieve method were introduced for assessing particle size distribution. Digital scanning was proposed; here, food particles are scanned and particle size, area, or weight are assessed using digital software (Eberhard et al., 2012; Eberhard, Schneider, Eiffler, Kappel, & Giannakopoulos, 2015; Mowlana, Heath, van der Bilt, & van der Glas, 1994). Other later methods include a spectrophotometer measurement of released dye or released glucose from fragmented test food particles (Escudeiro Santos, de Freitas, Spadaro, & Mestriner‐Junior, 2006; Ikebe, Morii, K‐i, Hazeyama, & Nokubi, 2005).

The degree of mixing and test‐food bolus shaping was suggested as an alternative. Color‐changeable chewing gum and two‐color wax or gum are used as test food (Liedberg & Owall, 1995; Prinz, 1999; Sugiura, Fueki, & Igarashi, 2009; Wada, Kawate, & Mizuma, 2017). Degree of mixing, measured by degree of color change, is assessed subjectively with a color scale or objectively with a colorimeter/scanner and digital software. Bolus shape is assessed with a bolus scale (Schimmel, Christou, Herrmann, & Muller, 2007; Wada et al., 2017).

To our knowledge, the measurement properties of the many different methods for assessing masticatory performance have never been critically appraised and reported. The objective of this systematic review is to identify studies that describe measurement properties of one or more methods intended to objectively assess masticatory performance and to establish their methodological quality by using a validated appraisal tool. Consequently, our systematic review intended to:

- Identify methods for objectively assessing masticatory performance;
- Evaluate measurement properties of the identified methods;
- Compare measurement properties of the identified methods;
- Identify adverse events during development or validation of methods that were studied.

## 2 METHODS

### 2.1 Design

This systematic review is reported as per PRISMA guidelines (Moher et al., 2015). The protocol was published and registered in the PROSPERO database (Ref: CRD42016037700; Elgestad Stjernfeldt, Wardh, Trulsson, Faxen Irving, & Bostrom, 2017). Some modifications of the original protocol were that the original aim, that is, “To evaluate psychometric properties (such as validity and reliability) of the identified methods”(Elgestad Stjernfeldt et al., 2017), was changed to “To evaluate measurement properties of the identified methods.” The rationale was to clarify that the review intents on evaluating measurement properties and not specifically psychometric methods. Moreover, the original protocol stated “… describes development of a method that objectively assesses clinical masticatory performance or evaluates measurement properties,” which was changed to “… describes a method that objectively assesses clinical masticatory performance and evaluates measurement properties in adults.” The changes were made because the study's aim was to evaluate measurement properties of various methods, rather than briefly describing them.

### 2.2 Information sources and literature search strategy

Five databases were searched from their inception up to January 2017: MEDLINE, Embase, Web of Science Core Collection, Cochrane, and Cinahl. In addition, Google Scholar identified more potentially relevant articles. The literature search was updated in December 2017 to identify any relevant articles published since the initial January 2017 search.

The overall search strategy was developed with librarians at Karolinska Institute University Library who ran the systematic literature searches. (Data S1).

### 2.3 Inclusion and exclusion criteria

The present systematic review focuses on full‐length articles published in English in scientific journals that contain measurement properties of methods used to assess masticatory performance in adults (ages ≥18). No restrictions occurred regarding type of timeframe for completing the assessments or type of study settings in which assessments were conducted.

The present systematic review excluded interview methods and self‐reported questionnaires; methods/instruments that subjectively assess masticatory performance; qualitative studies and case studies; expert opinions, editorial articles; animal studies; human studies (persons with severe oral health complications); and unavailable, full‐text studies.

### 2.4 Study selection

Two independent reviewers assessed all remaining titles and abstracts for eligibility. If this was insufficient for determining eligibility, then the full‐text articles were retrieved.

Full‐text articles were obtained from the remaining eligible abstracts. Two groups with two reviewers in each group independently judged each article for eligibility. One reviewer from each group independently screened the references lists of the all included articles for any additional relevant studies.

During each review phase, regular team meetings were held to discuss criteria. Several abstracts and articles were pilot‐tested to ensure agreement. Discussion and consensus resolved disagreements among reviewers.

### 2.5 Methodological quality assessment

The methodological quality of included studies was evaluated using a modular checklist, that is, Consensus‐based Standards for the selection of health Measurement INstruments (COSMIN; Terwee et al., 2012). COSMIN contains 12 boxes that are used to assess methodological quality of studies of measurement properties. Four domains are specified in COSMIN: validity, reliability, responsiveness, and interpretability with related measurement properties and their characteristics. For each of the measurement properties, the COSMIN consists of five to 18 items that cover methodological standards. In addition, each item is rated on a four‐point scale (i.e., poor, fair, good, and excellent; Terwee et al., 2012). By applying the lowest rating for each item in one box, an overall score is separately generated for each measurement properties. A study is rated as poor, fair, good, or excellent regarding methodological quality for each of the assessed measurement properties.

#### 2.5.1 Definitions

The COSMIN panel defines validity as “the degree to which an instrument truly measures the construct(s) it purports to measure” (HCWd, Terwee, Mokkink, & Knol, 2015; Mokkink et al., 2010). Criterion validity indicates degrees to which a measurement instrument's scores adequately reflect another method or instrument that is considered a gold standard. Criterion validity can only be assessed when a gold standard is available (HCWd et al., 2015; Mokkink et al., 2010). Construct validity is defined as “the degree to which the scores of an instrument are consistent with hypotheses.” Validation requires the formulation of specific hypotheses to acquire evidence that the instrument is measuring what it claims to measure (HCWd et al., 2015; Mokkink et al., 2010). Responsiveness is defined as “the ability of an instrument to detect change over time in the construct to be measured” (Mokkink et al., 2010). Reliability is defined as “the degree to which the measurement is free from measurement error” (Mokkink et al., 2010). Measurement error is defined as “the systematic and random error of a patient's score that is not attributed to true changes in the construct to be measured” (Mokkink et al., 2010).

### 2.6 Measurement properties quality

The qualities of measurement properties were established according to criteria developed by Terwee and colleagues (Terwee et al., 2007; Table 1). According to this framework, measurement properties are rated as positive, negative, or indeterminate. In the current systematic review, one reviewer rated all measurement properties, while review team confirmed the ratings.

**Table 1:** Quality criteria for rating the results of measurement properties, and evidence levels judged on the ratings of measurement properties^a^

Reliability		
Property	Rating	Quality criteria
Internal consistency	+	Cronbach's α(s) ≥ 0.70
	?	Cronbach's α not determined
	−	Cronbach's alpha(s) < 0.70
Measurement errors	+	Minimal important change > smallest detectable change, or minimal important change outside the limits of agreement
	?	Minimal important change not defined
	−	Minimal important change ≤ smallest detectable change, or minimal important change equals or inside limits of agreement
Reliability	+	Intraclass correlation/weighted kappa ≥ 0.70 or Pearson's *r* ≥ 0.80
	?	Neither Intraclass correlation/weighted kappa or Pearson's *r* determined
	−	Intraclass correlation/weighted kappa < 0.70 or Pearson's *r* < 0.80

Validity		
Property	Rating	Quality criteria
Construct validity – hypothesis testing	+	Correlation ≥0.50 with an instrument measuring the same construct, or ≥75% of the results in accordance with the hypotheses and correlation with related constructs is higher than with unrelated constructs
	?	Solely correlations determined with unrelated constructs
	−	Correlation <0.50 with an instrument measuring the same construct, or <75% of the results in accordance with the hypotheses, or correlation with related constructs is lower than with unrelated constructs
Content validity	+	The target population considers all items in the questionnaire to be relevant, or considers the questionnaire to be complete
	?	No target population involvement
	−	The target population considers all items in the questionnaire to be irrelevant, or considers the questionnaire to be incomplete
Criterion validity	+	Convincing arguments that gold standard is “gold” and correlation with gold standard ≥ 0.70
	?	No convincing arguments that gold standard is “gold” or doubtful design or method
	−	Correlation with gold standard < 0.70, despite adequate design and method
Cross‐cultural validity	+	Original factor structure confirmed, or no important differential item functioning between language versions
	?	Confirmatory factor analysis not applied and differential item functioning not assessed
	−	Original factor structure not confirmed, or important differential item functioning found between language versions
Structural validity	+	Factors explain ≥50% of the variance
	?	Factors variance not mentioned
	−	Factors explain <50% of the variance

Responsiveness	
Rating	Quality criteria
+	Correlation with an instrument measuring the same construct ≥0.50, or at least 75% of the results are in accordance with the hypotheses or area under the receiver operating characteristics (ROC) curve, and correlation with related constructs is higher than with unrelated constructs
?	Solely correlations determined with unrelated constructs
−	Correlation with an instrument measuring the same construct <0.50 or <75% of the results are in accordance with the hypotheses or area under the ROC curve, or correlation with related constructs is lower than with unrelated constructs

Evidence levels	
Level	Criteria
Strong	Consistent findings, multiple studies of good methodological quality OR ≥ 1 study of excellent methodological quality
Moderate	Consistent findings, multiple studies of fair methodological quality OR ≥ 1 study of good methodological quality
Limited	One study of fair methodological quality
Conflicting	Conflicting findings
Unknown	Only studies of poor methodological quality

*Note*. Rating: (+) = positive; (?) = indeterminate; (−) = negative.

a Adapted from: Dobson et al., Ostheoarthritis and Cartilage, 2012 and Terwee et al., J Clin Epidemiol 2007.

### 2.7 Evidence levels

Data synthesis for each methods for assessing masticatory performance occurred by combining methodological quality of included studies and measurement properties quality Table 1. First, all studies were quality assessed separately, and then for each method. Studies that evaluated the same method were given an individual score, and the results were then pooled in an overall evidence synthesis. The level of evidence was synthesized across the studies with an overall conclusion, namely, unknown, conflicting, limited, moderate, or strong level of evidence. Table 1 describes rating criteria.

## 3 RESULTS

**Figure 1:**
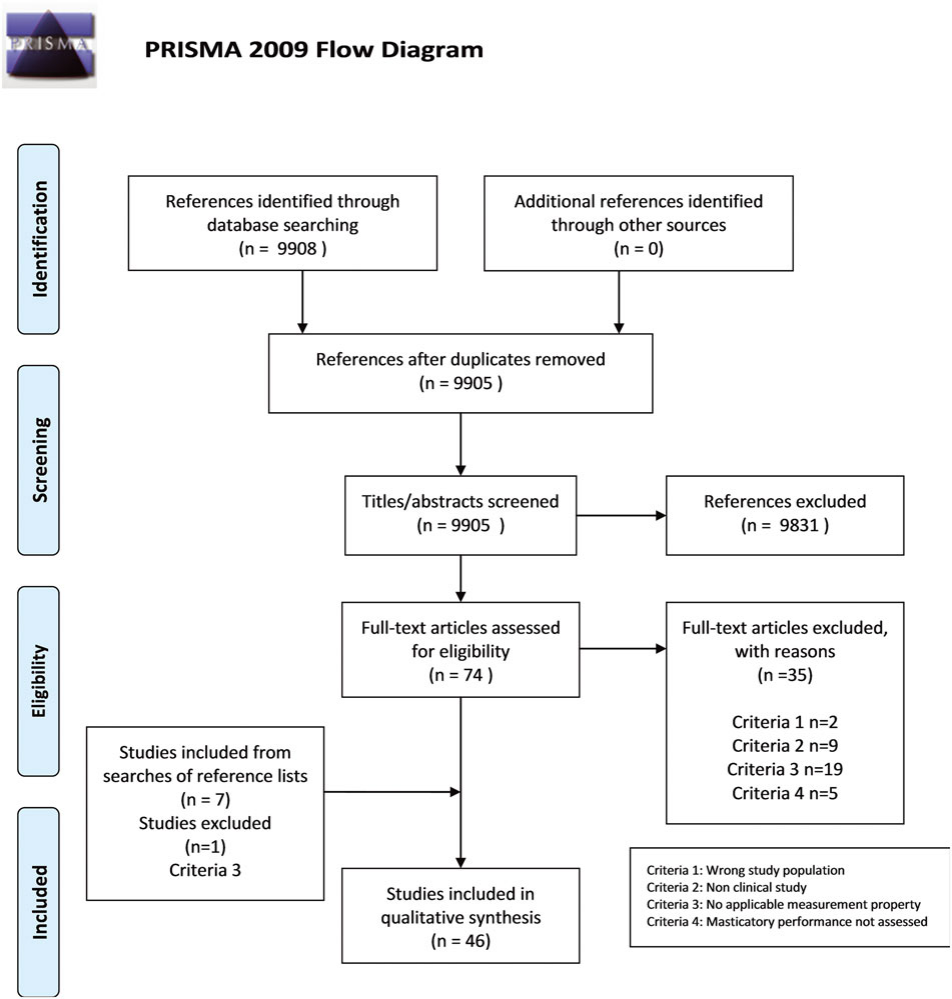
Flow Diagram

The PRISMA diagram (Figure 1) illustrates the inclusion process of articles. The present systematic review included 46 articles that represent 46 studies (Table 2). The updated literature search in December 2017 yielded no new eligible articles. Data S1 lists all excluded full‐text articles. Data S2 lists non‐English studies that were identified during screening of references, but were not included.

**Table 2:** Included studies

		Comminution tests		
Study, first author	Study objective	Measurement property	Participants and age (range years or mean age**)**	Materials and methods
Khoury‐Ribas et al. (2017)	Assessment of Optosil Plus® and sieve as a method to assess MP. Optozeta as a test food compared with Optosil.	Validity (hypotheses testing), reliability	*n* = 24, 11 excluded from *n* = 35 (12 M, 23 F)*n* = 10, 5 excluded from *n* = 15,participated in retest after 1–2 weeks (4 M, 11 F) 19–77 Retest: mean age, 34.	Method: Comminution. Test food: Optozeta tablets. Test food particles separated by a stack of eight sieves. Activity: CS = 20 in five trials in two assays with both Optosil and Optozeta tablets. Performance measure: X50 = theoretical sieve aperture value were 50% of particle wgt. can pass. Broadness = b.
Sanchez‐Ayala et al. (2016)	Evaluation of encapsulated fuchsine beads as a method to assess MP.	Validity (criterion validity), reliability, measurement error	*n* = 20 (5 M, 15 F)23.3 ± 0.7	Method: Comminution. Test food: Encapsulated fuchsine beads. Fuchsine dye, released from chewing, quantified with spectrophotometer. Activity: CS = 20. Portion: one capsule. Three tests with 1 week interval and two additional tests Performance measure: Absorbance units (AU). Masticatory performance proportional to AU.
Eberhard et al. (2015)	Comparison of optical scanning of fragmented test food particles to sieve method with 10 sieves.	Validity (criterion validity)	*n* = 16, (12 male, 4 female). 68.6 ± 9.34	Method: Comminution. Optocal as test food. Particles scanned and analyzed in a digital image processing software. Flatbed scanner and ImageJ and Xnview software. Activity: Optocal CS = 15, 40. Portion: 17 cubes per test. Performance measure: Particle area volume and wgt.. 10 wgt. values per sample. Median particle size (X_50_).
Nokubi et al. (2013)	Assessment of a visual scoring scale, 1–10, to assess MP with gummy jelly as test food. Visual scoring scale compared with objective glucose concentration released from jelly, which is proportional area size of test food particles.	Validity (hypotheses testing), reliability	*n* = 1, individual comminuted 50 test food gummy jellies. *n* = 50 raters (26 M, 24 F). Examiners 33.4 ± 10.6	Method: Comminution. Test food: Gummy jelly. Visual scoring scale, score 1–10, to assess glucose concentration released from a gummy jelly test food and MP. Activity: CS not defined. gummy jellies and released glucose concentration ordered into 10 different groups according to visual scale. Performance measure: 10‐stage visual scale for rating MP of a comminuted gummy jelly.
Sanchez‐Ayala et al. 2014	Assessment of Optosil Comfort®as an artificial test food for MP evaluation using sieve method	Reliability, measurement error	*n* = 20 (5 M, 15 F) 23.3 ± 0.7	Method: Comminution. Optosil comfort as test food. Particles separated by sieve machine (simple‐, double‐, multiple‐sieve method) with a stack of up to 10 sieves w/apertures ranging 0.5 to 5.6 mm. Activity: CS = 20. Portion: 17 cubes (3.4 g). Three tests with 1 week interval and two additional tests. Performance measure: Particle wgt. on each sieve.Single sieve: Wgt. (%) of particles passing through each sieve.Double sieve: Wgt. (%) of particles retained on first and second sieve/total particle wgt..Multiple sieve: X_50_ = theoretical sieve aperture value were 50% of particle wgt. can pass. Broadness = b
Eberhard et al. (2012)	Comparison of optical scanning of fragmented test food particles to sieve method with 10 sieves.	Validity (criterion validity)	*n* = 16, (12 male, 4 female) *n* = 20 (10 M, 10 female) 24 ± 2	Method: Comminution. Optosil comfort as test food. Particles scanned and analyzed in digital image processing software. Flatbed scanner and ImageJ software and Xnview software. Activity: Optosil Comfort CS = 15, Portion: 17 cubes per test. Performance measure**:** Particle area volume and wgt.. 10 wgt. Values per sample. Median particle size (X50).
Woda et al. (2010)	Assessment of MP with masticatory normative indicator.	Validity (hypotheses testing)	Young dentate = 12, aged denture wearers *n* = 14, aged dentate *n* = 14 Young dentate = NR, aged denture = 68.1 ± 7.2, aged dentate = 68.8 ± 7.0	Method: Comminution. Test food: ground nuts and carrots. Aged denture/dentate: Particles separated by stack of seven sieves. Young: Particles separated by scanning.Activity: Chewing until deglutition. Performance measure: Mean d_50_ distribution. Masticatory normative indicator: Median particle size of carrot 4,0 mm (cut off point).
Fauzza & Lyons (2008)	Assessment of alginate as a test food to assess MP in denture wearers.	Responsiveness	*n* = 20 (10 M, 10 female) 64–83	Method: Comminution. Alginate/hydrocolloid as test food. Particles separated by gravimetric sieve. Mesh sizes: 2.00, 1.70, 1.40, and 1.00 mm. Activity: Test performed first with old denture. CS = 10, 20. Tests repeated three times. 11 individuals (*n* = 11) retested with new denture after 2–4 weeks. Performance measure: Total particle wgt. for each sieve/total particle wgt. collected from all sieves (%).
Felicio et al. 2008	Evaluation of encapsulated fuchsine beads as a method to assess MP.	Validity (hypotheses testing), reliability.	*n* = 19 9 M, 10 F 18–28	Method: Comminution. Test food: Capsules containing fuchsine beads. Amount of fuchsine released. After chewing analyzed with spectrophotometer. Activity: Capsules masticated for 20 s in free habitual manner, then repeated on left and right side. Performance measure: Amount of fuchsine released, μg/ml.
Lujan‐Climent et al. (2008)	Assessment of Optosil Plus®& sieve as a method to assess MP.	Reliability, measurement error	*n* = 100 (29 M, 71 F)Reliability study *n* = 9 gender NR. 20.3–47.9 Reliability study: NR	Method: Comminution. Test food: Optosil Plus. Test food particles separated by a stack of 8 sieves.Activity: CS = 20, repeated five times.Reliability study: Trial repeated after 2–4 weeks. Performance measure: X_50_ = theoretical sieve aperture value were 50% of particle wgt. can pass. Broadness = b
Escudeiro Santos et al. (2006)	Evaluation of encapsulated fuchsine beadsas a method to assess MP	Reliability	*n* = 10 (5 M, 5 F) 25–30	Method: Comminution. Test food: Capsules containing fuchsine beads. Amount of fuchsine releasedafter chewing analyzed with spectrophotometer. Activity: three capsules masticated for 20 s. Three tests conducted. Performance measure: Amount of fuchsine released, μg/ml.
Ikebe et al. 2005	Evaluation of gummy jelly as a test food to assess MP.	Validity (hypothesis testing)	Gender and age NR	Method: comminution. Test food: gummy jelly. Fragmentation of gummy jelly particles can be calculated by concentration of released glucose. MP can then be assessed by evaluating the degree of fragmentation of test gummy jelly. Activity: Subjects instructed to chew one block of jelly on preferred side and pace. Performance measure: Glucose extraction (mg/dl) as a measure of MP.
Kobayashi et al. (2006)	Evaluation of gummy jelly as a test food to assess MP.	Validity (criterion validity)	*n* = 20 (10 M, 10 F) “All in their 20s”	Method: Comminution. Test food: gummy jelly. Glucose extraction from gummy jelly during chewing collected w/a filter. Filtrate measured chromatically and quantitatively by glucose‐oxidase method. Activity: CS = 10, 20, 30 on habitual side. Performance measure: Glucose extraction (mg/dl) as a measure of MP.
Shiga et al. (2006)	Assessment of MP with gummy jelly as test food and blood glucose meter to measure glucose concentration released after chewing	Validity (hypothesis testing)	*n* = 20 (10 M, 10 F) Mean age 24.6	Method: Comminution. Test food: gummy jelly. Glucose extraction from gummy jelly during chewing. Glucose concentration measured with blood glucose meter. Activity: Subjects instructed to chew one block of jelly on preferred side and pace for 20s. Performance measure: Glucose extraction (mg/dl) as a measure of MP.
Ohara et al. (2003)	Assessment of alginate as a test food and sieve method to assess MP.	Validity (hypotheses testing), reliability	*n* = 30, two groups, A and B. Group A: 10 M, 5 F.Group B: 10 M, 5 F. Group A: 23–36Group B: 24–35	Method: Comminution. Alginate/hydrocolloid as test food. Particles separated by sieve.10 mesh sizes: 4.75, 4.00, 2.80, 2.00, 1.70, 1.40, 1.18, 1.00, 0.85, and 0.71 mm. Activity: CS = 5, 10, 20, 25, 30, 35. Portion: one piece of hydrocolloid material.Group B: Test conducted three times during 1 day. Repeated on three different days, with 1 week interval. Performance measure: Test if there is a linear relationship between CS and particle wgt. and numbers on each sieve.
Huggare (1997)	Evaluation of color bindingtablets to assess MP. Dye concentration Measured with spectrophotometer	Validity (hypotheses testing), reliability	*n* = 4, two groups: natural dentition: *n* = 2 (one male, one female).Partial denture: *n* = 2 (1 male, 1 female). Natural dentition: 22 and 25Partial denture: 55 and 60.	Method: Comminution. Test food: tablets incorporating a color binder. Particles separated through filter of glass wool. Particles placed in water‐soluble dye. Particles absorb dye. Spectrophotometer measures absorption/concentration of dye in solution. Activity: CS = 10. Portion: four tablets. Test repeated on same occasion and after 1 week. Partial denture group conducted test with and without denture. Performance measure: Concentration of dye in solution decreases in proportion to particle size area (difference in light absorption (ΔA). Particle area measure of test food breakdown (masticatory efficiency)
Mowlana et al. (1994)	Assessment of optical scanning as a method to analyze fragmented test food particles, using flatbed scanner and digital image software.	Validity (hypotheses testing)	*n* = 6 (3 M, 3 F) 21–29	Method: Comminution. Test food almonds. Particles scanned and analyzed in digital image processing software. Activity: CS = 1, 4, 8, 16, 32. Performance measure: Cumulative volume distribution of particles. Theoretical sieve aperture values were 50% of particle volumes can pass. Broadness = b, distribution of particles sizes.
Slagter et al. (1993)	Comparison of Optocaland Optosil as a test food with sieve test.	Validity (hypotheses testing), reliability	*n* = 14, two groupsNatural dentition: n = 7, gender NRComplete dentures: *n* = 7, gender NR. Natural dentition: 33–70Complete dentures: 50–71	Method: Comminution. Test food: Optocal and Optosil. Test food particle separated by a stack of 10 sieves. Activity: CS = 10, 20, 40, 60, 80. Test conducted two times. Performance measure: Theoretical sieve aperture values were 50% of particle volumes can pass, X_50_.
Mahmood et al. (1992)	Assessment of an image analyzer, Magiscan 2, as a method to analyze fragmented test food.	Validity (hypotheses testing), responsiveness, reliability	*n* = 30, gender = NRThree groupsNew complete dentures: *n* = 10 (before and after new denture)Natural dentition: *n* = 10Old complete dentures: *n* = 10 Age NR	Method: Comminution. Test food carrot. Test food particles analyzed w/image analyzer. Activity: CS = 20 and swallowing threshold. Test repeated after 6 months with patients provided with new dentures Performance measure: Particle measurements: Particle area. Particle length. Particle breadth.
Gunne (1985)	Evaluation of gelatin hardened by formalin as test food to assess MP. Method compared to sieve method with almonds.	Validity (hypotheses testing)	*n* = 45 Dentate n = 11, 0 M 11 F. Partial dentures *n* = 11, 5 M 6 F Complete dentures *n* = 13, 6 M 7 F. Dentate *n* = 10, 0 M 10 F. Dentate 20–26 Partial dentures 51–68 Complete denture 56–77 Dentate 18–23	Method: Comminution. Test food: Gelatin hardened by formalin. Test food particles absorb dye. Amount of dye absorbed in relationship to particle size of fragmented test food and MP Activity: 22 mm test‐cubes. Performance measure: Mean particle area of fragmented gelatin test food (cm^2^).
Kapur et al. (1964)	Assessment of carrot as test food to evaluate MP in dentures wearers. Single sieve.	Reliability	Test1: *n* = 22, denture wearers. Gender NR Test2: n = 22, denture wearers. Gender NR Age NR.	Method: Comminution. Raw carrot as test food. Test food particles separated by a stack of seven sieves. Activity: Test 1: CS = 40. Test food raw carrot. Total three tests under same sitting. Test 2: MP‐test performed once per week, for 12 weeks on same chewing side. MP‐test method not described. CS = NR Performance measure: Volume of test food passing through a given sieve/total volume of recovered test food (%).

		Mixing tests		
Study, first author	Study objective	Measurement property	Participants and age (range years or mean age)	Materials and methods
Silva et al. (2018)	Assessment of color mixing with w/color scale and digital image software.	Reliability, measurement error	Complete denture wearers *n* = 75 (24 M, 51 F). 67.1 ± 8.5	Method: Mixing ability. Two‐colored chewing gum. Color change assessed visually with color scale and digital image software. Activity: CS = 5, 10, 20, 30, 40, 50 Performance measure: Color scale: Score 1–5. Image software: Variance of hue (VOH)
Wada et al. (2017)	MP assessed with color changing chewing gum, Masticatory Performance Evaluating Gum XYLITOL and digital image software.	Validity (hypothesis testing)	*n* = 30 (12 M, 18 F) 81.6 ± 8.6	Method: Mixing ability. Color changeable chewing gum. Assessment of color change with a colorimeter and digital image software. Activity: Participants chewed gum for 120 s. Performance measure: a* = color change between green and red according to CIELAB color system.
Vaccaro et al. (2016)	Assessment of color mixing with a digital software on photographed wafer. Gum: red and white.	Validity (hypotheses testing)	Participants: *n* = 250 (120 M, 130 F)Chewing gum samples *n* = 2000 M 25 ± 6.8 F 25 ± 5.8	Method: Mixing ability. Two‐colored chewing gum. Assessment of color mixing with digital image processing software, MATLAB 2015b. Activity: CS = 3, 6, 9, 12, 15, 18, 21, 25. Performance measure: Variance of the histogram of the Hue(VhH).
Schimmel et al. (2015)	Assessment of color mixingw/digital software, ViewGum, on scanned wafer. Gum1 azure and pink, Gum 2 blue and red, Gum 3 green and dark violet. Subjective assessment of gum bolus. Scores 1–5 and assessment of color mixing of flattened gum wafer.	Validity (hypotheses testing), reliability	*n* = 35 divided into 2 groups. Dentate group *n* = 20 (10 M, 10 F)Implant overdenture *n* = 15 (5 M, 10 F). Dentate: 30.3 ± 6.7Implant overdenture: 74.6 ± 8.3	Method: Mixing ability. Two‐colored chewing gum (three different). Color change and bolus shape of two‐colored chewing gum (mixing ability) assessed visually with color scale and digital image software ViewGum. Activity: Test 1: Dentate group. CS = 5, 10, 20, 30, 50.Test 2: Both groups. CS = 20 Performance measure: Color/bolus scale: Score 1–5Image software: Variance of hue (VOH)
Endo et al. (2014)	Assessment of color mixingwith digital software, Adobe Photoshop CS3®, on scanned wafer. Gum blue and red.	Reliability	*n* = 31, 16 M, 15 F M 24.8 ± 2.3F 23.8 ± 1.5	Method: Mixing ability. Test food: two‐colored chewing gum. Subjective assessment: bolus shape and color mixing. Wafer color mixing. Objective assessment: Computerized measurement of color mixing in wafer Activity: CS: 5, 10, 20, 30, 50. Test repeated on different day with same subjects. Performance measure: Bolus index score: 1–5.Shape index score: 1–4.Unmixed pixels/total pixels (%) of scanned wafer.
Hama et al. (2014a)	Assessment of color change inMasticatory Performance Evaluating Gum XYLITOL to rate MP.	Validity (hypotheses testing), reliability	Two testsTest 1: *n* = 10, 70% M 30% FTest 2: *n* = 8942 dentate (52% M, 48% F), 47 edentulous (43% male Test 1: 27.7 ± 1.5Test 2:Dentate: 26.8 ± 2.6Edentulous: 74.9 ± 10.5, 57% F)	Method: Mixing ability. Color changing chewing gum. Color change of gum assessed with colorimeter and CIELAB color system (a*, b* L*).ΔE and relationship with #CS. MP assessed dentate/edentulous groups. Activity: Test 1: three participants CS = 20, 240, 60, 80, 100, 120, 160, 200, 300, 400, 500 600. Repeated three times.Seven participants, CS = 20, 40, 60, 80, 100, 120, 160, 200. Repeated five times. Test 2. CS = 100, 1 CS/second. Performance measure: Color change (ΔE) before and after chewing gum.
Hama et al. (2014b)	Assessment of MP with color change of gum. Color scale, scores 1–11, to assess color change in color‐changeable chewing gum. Color scale compared with colorimeter and CIELAB color system as golden standard. (L* a* b*)	Validity (criterion validity), reliability	*n* = 18, (6 dentists, 6 dental students, 6 elderly individuals) Dentists: 25–27Dental students: 21–23Elderly individuals: 68–84	Method: Mixing ability. Evaluation of 32 prechewed gums with a developed color scale. Activity: Same examination repeated three times. Performance measure: Color scale score: 1–11
Halazonetis et al. (2013)	Assessment of color mixingwith digital software, ViewGum, on scanned wafer. Gum: azure and pink.	Validity (hypotheses testing), measurement error	*n* = 20, 11 M, 9 F 27.5 ± 3.4	Method: Mixing ability. Test food: two‐colored chewing gum. Degree of color mixing (SDHue) assessed with image analysis software. Relationship SDHue and CS. SDHue decrease with increasing CS. Activity: CS: 5, 10, 20, 30, 50. Performance measure: SD of hue of scanned bolus.
Weijenberg et al. (2013)	Assessment of color mixing with a digital software on photographed wafer. Gum soft pink and blue.	Validity (hypotheses testing), reliability	Test 1: *n* = 14 (7 M, 7 F)Test2: *n* = 10 (4 M, 6 F)Test 3: *n* = 13(13 F) Test 1: 19–63Test 2: 20–49Test 3: 21–31	Method: Mixing ability. two‐colored wax. Assessment of color mixing w/a digital image software, Mathematica. Activity: Test 1: CS = 5, 10, 15, 20, 25, 30Tests 2 and 3: 20 s duration Performance measure: Pixel intensity, DiffPix, a scale between 0 and 1.
van der Bilt et al. (2012)	MP assessed with two‐colored wax and Mixing ability test (MAI). MAI rated by visual assessment. Compared with optical scanning and digital image analysis.	Validity (hypothesis testing), reliability	*n* = 60, three groups. Natural dentition *n* = 20 (10 M, 10 F), Dentures/implant overdentures *n* = 20 (11 M, 9 F), Full denture n = 20 (10 M, 10 F). Natural dentition: 58.2 ± 4.4Denture/implant overdentures: 62.2 ± 7.3Full denture: 60.5 ± 9.0240 digital images analyzed by five examiners.	Method: Mixing ability. two‐colored wax. Assessment of color change w/optical scanning & digital image software, Adobe Photoshop CS3 MAI by visual assessment. **Activity:** CS = 5, 10, 15, 20 **Performance measure:** MAI rated by visual assessment on a five‐point scale. Digital image analysis: SD of intensity of distribution red and blue color.
Abe et al. 2011	Two‐colored rice and uirou (rice cake) mixing assessed with *v*ideo endoscopic in the oropharynx &digital image software.	Validity (hypotheses testing)	*n* = 10 (5 M, 5 F) 27.1 ± 1.7	Method: Mixing ability. Test food: two‐colored rice and uirou (rice cake) Activity: CS = 10, 15, 20, 30 and under instruction to “chew normally” and “chew well”. Test performed three times. Performance measure: Bolus formation index (BFI) A = total area of bolus (in pixels).W = white area of bolusBFI = (A‐W)/AProportion of non‐white area to the entire food bolus area = BFI.
Kamiyama et al. (2010)	Assessment of color scale used to rate color change of masticatory Performance Evaluating Gum XYLITOL. Compared with colorimeter using CIELAB color system, (a*)	Validity (criterion validity), reliability	*n* = 18, (6 dentists, six undergraduate students, six elderly individuals) Dentists: 28–31Students: 22–27Elderly individuals: 70–80	Method: Mixing ability. Color changing chewing gum. Color change assessment rating with color scale (mean color score).Activity: Evaluation of 30 prechewed color changeable chewing gum and color scale. Repeated three times. Performance measure: Color scale: five color chart with a 100 mm long visual analog scale.
van der Bilt et al. (2010)	Assessment of color mixing of two‐colored gum with digital software. Method compared with GS, comminution/sieve test. Test food Optosil and Optocal.	Validity (hypotheses testing)	*n* = 40, two groups. Elderly n = 20 (5 M, 15 F). Young *n* = 20 (6 M, 14 F). Elderly: 72.1 ± 7.5Young: 24.0 ± 4.2	Method: Mixing ability. two‐colored gum. Color change assessed w/digital image software, Adobe Photoshop CS2®, on scanned wafer. Activity: CS = 10, 20 Performance measure: The SD of intensity distribution (color) = mixing index.
Speksnijder et al. (2009)	MP assessed with two‐colored wax and mixing ability test that assesses color mixing. Assessment of color mixing with digital image software. Compared with comminution/sieve test.	Validity (hypotheses testing)	*n* = 60, 3 groups. Natural dentition *n* = 20 (10 M, 10 F), Dentures/implant overdentures *n* = 20 (11 M, 9 F), Full denture n = 20 (10 M, 10 F). Natural dentition: 58.2 ± 4.4Denture/implant overdentures: 62.2 ± 7.3Full denture: 60.5 ± 9.0	Method: Mixing ability. Two‐colored wax. Assessment of color change with optical scanning and digital image software Adobe Photoshop CS3. Activity: CS = 5, 10, 15, 20 Performance measure: The SD of intensity of distribution red and blue color.
Sugiura et al. (2009)	Assessment of color mixing and bolus shape and MP evaluated w/the MAI and two‐colored wax.. Compared MAI with comminution/multiple sieve test, test food peanuts, and gummy jelly	Validity (hypotheses testing), measurement error.	*n* = 72, 2 groups.Natural dentition, *n* = 32 (18 M, 14 F).Partially edentulous, *n* = 40 (18 M, 22 F). Natural dentition: 25.1 ± 2.8Partially edentulous: 65.5 ± 9.1	Method: Mixing ability. Two‐colored wax. Mixture of colors analyzed with digital image and LUSEX‐FS image analyzer. Activity: CS = 10. Test performed 3 times. Performance measure: Mixing ability index.
Ishikawa et al. (2007)	Color change of Masticatory Performance Evaluating gum XYLITOLColor change assessed with colorimeter. Responsiveness of the method is compared with a comminution/sieve test and two subjective tests.	Responsiveness	*n* = 26, edentulous patients. 63–92	Method: Mixing ability. Color changing chewing gum. Color change assessment rating with color scale (mean color score of the three tests) compared to colorimeter using CIELAB color system (a*).Activity: Test performed first w/old denture, then w/new denture. Mean time interval 4 weeks. CS = 100. Test repeated three times. Performance measure: Colorimeter: a* = mean value of color red.
Schimmel et al. (2007)	Assessment of color mixingw/digital software, Adobe Photoshop Elements®, on scanned wafer. Gum azure and pink. Subjective assessment of gum bolus, scores 1–5 and assessment of color mixing of flattened gum wafer.	Validity (hypotheses testing), reliability	*n* = 20, 9 M, 11 F. 27.5 ± 3.4.	Method: Mixing ability. Two‐colored chewing gum. Subjective assessment: bolus shape and color mixing. Wafer color mixing.Objective assessment: Computerized measurement of color mixing in wafer. Activity: CS = 5, 10, 20, 30, 50. *n* = 15 repeated test on different day Performance measure: Bolus index score: 1–5.Shape index score: 1–5.Computerized measurement: Unmixed fraction (pixels).
Asakawa et al. (2005)	Is a two colored wax and the Mixing Ability Index (MAI) able to detect difference of masticatory function before and after denture treatment?	Responsiveness	n = 32, 7 M, 25 F. 65 ± 6.5	Method: Mixing ability. Test food: two‐colored wax cube. Color mixing and shape of two colored wax cube, MAI. Assessed with image analyzer Activity: Test performed first with old denture, then with new denture. Mean time interval between test = 27, 4 weeks. CS: 10. Performance measure: Mixing ability index
Sato et al. (2003)	Assessment of color mixing and bolus shape and MP evaluated with the MAI and two‐colored wax. Compared MAI with comminution/sieve test as golden standard, comminuting ability index (CAI).	Validity (hypotheses testing), reliability	Two testsTest 1: *n* = 44, divided into three groups. Group A (7 M, 4 F), group B (2 M, 18 F), and group C (5 M, 8 F).Test 2: *n* = 3 (three dentate subjects), gender NR Test 1: A 25–32. B 42–75, C 55–77Test 2: NR	Method: Mixing ability. two‐colored wax. Color change and bolus shape assessed with digital image analysis and Mixing ability index. Activity: Test 1: CS = 5, 7, 10, 15, 20, & 30.Test 2: CS = 5, 7, 10, 15, 20, and 30. Repeated three times. Also test repeated two times with time interval 1 week. Lastly, one test CS = 10 repeated three times. Performance measure: Mixing ability index
Prinz (1999)	MP assessed with two‐color chewing gum. Digital photograph of flattened gum analyzed with image processing technique.	Validity (hypotheses testing), measurement error.	*n* = 10 (7 M, 3 F) Age NR	Method: Mixing ability. two‐colored chewing gum. Analyzed with graphics unbiased measurement system. Image processing techniques Activity: CS=“Between 2 and 30”A further set of 10 subjects chewed CS = 5, 20, 30. Performance measure: Digital image processing: Low contrast, High contrast, Polarity, Fragmentation, Blending, Spatial frequency, Nearest neighbor analysis. Subjective: Ranking of two different sets with 10 samples. One set with gum bolus and one set with flattened gum.
Hayakawa et al. (1998)	Assessment of colorchangeable chewing gum and color scale for assessment of MP.	Validity (hypotheses testing)	Two testsTest 1: *n* = 18. (16 M, 2 F)Test 2: *n* = 6, (5 M, 1 female) Test1: 20–28Test 2: 21–25	Method: Mixing ability. Color changing chewing gum. Color change assessed with spectrophotometer and color scale.Activity: Test 1: CS = 5, 15, 30, 45. Test 2: CS = 5, 15, 30, and 45. Performance measure**:** Color change Spectrophotometer: a* = mean value of color red.Color scale: Scores 1–4.
Matsui et al. (1996)	Evaluation of color changeable chewing gum ax a test food to assess MP.	Validity (hypotheses testing), measurement error	Two testsTest 1: *n* = 5, 5 M.Test 2: *n* = 30, 15 dentate (8 M 7 F), 15 edentulous (8 M, 7 F). Test 1: 25–28Test 2: Dentate 22–35, edentulous 63–79.	Method: Mixing ability. Color changeable chewing gum. Color changed assessed w/colorimeter. Spectrophotometer: a*value. a* divided into 4 groups. Activity: Test 1: CS = 10, 30, 50, 70, 90. Test repeated three times. Test 2: CS = 50. Test repeated three times. Performance measure: Spectrophotometer: a* = value of color red. a* divided into four groups.
Liedberg & Owall (1995)	Subjective assessment of color mixing and bolus shaping with color scale 1–5, and bolus scale 1–5.	Reliability	Two testsTest 1: *n* = 25, 5 M, 20 FTest 2: *n* = 20, gender: NR Test 1: 32–82Test 2: Age NR	Method: Mixing ability. Two‐colored chewing gum. Color change assessed with color scale. Bolus shape assessed w/bolus index. Activity: Test 1: CS = 10, 20, 40, 60, 80, and 100.Test 2: CS = 10. Chewed 15 gums (five right sides, five left side, five habitual). Test repeated on separate occasion. Performance measure: Color index: score 1–5Bolus index: 1–5

		Other methods		
Study, first author	Study objective	Measurement property	Participants & Age (range years or mean age)	Materials and methods
Goto et al. (2016)	Assessment of odor intensity after chewing chewing gum test food, as a measurement of MP. Measured with a portable odor sensor device. Compared with gummy jelly test food.	Validity (hypotheses testing), measurement error	*n* = 20 (12 M, 8 F) 26.9 ± 3.26	Method**:** Odor intensity. Test food: Chewing gum Clorets XP®. Odor intensity after chewing test food measured w/portable odor sensor device (OMX‐SR). Activity: Chewing duration: 10, 20, 30, and 40 s.Odor intensity measured immediately, 1, 5 and 10 min after chewing the gum. Test repeated three times. Performance measure: Odor intensity value(mean and SD).
Ikebe et al. (2005)	Assessment of MP with Eichner index. Compared with glucose releasing gummy jelly test for assessment of MP.	Validity (hypotheses testing)	*n* = 1288 (640 M, 648 F) 66.2 ± 4.2	Method: Posterior occluding contactsare registered in a dental examination and divided into 10 m subgroups: A1, 2, 3 = occluding contacts in all four posterior support zones.B1, 2, 3 = occluding contacts in one to three support zones or contact in anterior region only.C1, 2, 3 = no occlusal contacts at all. Activity: Patients were grouped into 10 subgroups by posterior occlusal contacts according to the Eichner index. Performance measure: Number of occluding posterior support zones.

### 3.1 Measurement properties

The majority of the studies (*n* = 32, 70%) were rated as poor or fair (Abe, Furuya, & Suzuki, 2011; Asakawa, Fueki, & Ohyama, 2005; Felicio, Couto, Ferreira, & Mestriner Junior, 2008; Eberhard et al., 2012; Eberhard et al., 2015; Endo et al., 2014; Fauzza & Lyons, 2008; Goto et al., 2016; Halazonetis, Schimmel, Antonarakis, & Christou, 2013; Hama, Kanazawa, Minakuchi, Uchida, & Sasaki, 2014a; Hama, Kanazawa, Minakuchi, Uchida, & Sasaki, 2014b; Hayakawa, Watanabe, Hirano, & Nagao, 1998; Huggare, 1997; Ishikawa, Watanabe, Hayakawa, Minakuchi, & Uchida, 2007; Kamiyama, Kanazawa, Fujinami, & Minakuchi, 2010; Khoury‐Ribas, Ayuso‐Montero, Rovira‐Lastra, Peraire, & Martinez‐Gomis, 2017; Kobayashi, Shiga, Arakawa, & Yokoyama, 2006; Lujan‐Climent et al., 2008; Mahmood, Watson, Ogden, & Hawkins, 1992; Matsui et al., 1996; Mowlana et al., 1994; Nokubi et al., 2013; Ohara, Tsukiyama, Ogawa, & Koyano, 2003; Prinz, 1999; Sato et al., 2003; Schimmel et al., 2007; Schimmel et al., 2015; Shiga, Kobayashi, Arakawa, Yokoyama, & Unno, 2006; Slagter, Bosman, & Van der Bilt, 1993; Sugiura et al., 2009; Wada et al., 2017; Weijenberg et al., 2013) mainly to small sample sizes. Only a minority of the studies (*n* = 4, 9%) presented sample size calculations (Khoury‐Ribas et al., 2017; Sanchez‐Ayala et al., 2016; Sanchez‐Ayala, Vilanova, Costa, & Farias‐Neto, 2014; Wada et al., 2017).

Different domains and measurement properties were reported, of which, the most common was to report the validity of the method for assessing masticatory performance (*n* = 36 studies; Abe et al., 2011; Felicio et al., 2008; Eberhard et al., 2012; Eberhard et al., 2015; Goto et al., 2016; Gunne, 1985; Halazonetis et al., 2013; Hama et al., 2014a; Hama et al., 2014b; Hayakawa et al., 1998; Huggare, 1997; Ikebe et al., 2005; Ikebe, Matsuda, Murai, Maeda, & Nokubi, 2010; Kamiyama et al., 2010; Khoury‐Ribas et al., 2017; Kobayashi et al., 2006; Mahmood et al., 1992; Matsui et al., 1996; Mowlana et al., 1994; Nokubi et al., 2013; Ohara et al., 2003; Prinz, 1999; Sanchez‐Ayala et al., 2016; Sato et al., 2003; Schimmel et al., 2007; Schimmel et al., 2015; Shiga et al., 2006; Slagter et al., 1993; Speksnijder, Abbink, van der Glas, Janssen, & van der Bilt, 2009; Sugiura et al., 2009; Vaccaro, Pelaez, & Gil, 2016; van der Bilt, Mojet, Tekamp, & Abbink, 2010; van der Bilt, Speksnijder, de Liz Pocztaruk, & Abbink, 2012; Wada et al., 2017; Weijenberg et al., 2013; Woda et al., 2010). Construct validity or hypotheses testing was frequently described (*n* = 30 studies; Abe et al., 2011; Felicio et al., 2008; Goto et al., 2016; Gunne, 1985; Halazonetis et al., 2013; Hama et al., 2014a; Hayakawa et al., 1998; Huggare, 1997; Ikebe et al., 2005; Ikebe et al., 2010; Khoury‐Ribas et al., 2017; Mahmood et al., 1992; Matsui et al., 1996; Mowlana et al., 1994; Nokubi et al., 2013; Ohara et al., 2003; Prinz, 1999; Sato et al., 2003; Schimmel et al., 2007; Schimmel et al., 2015; Shiga et al., 2006; Slagter et al., 1993; Speksnijder et al., 2009; Sugiura et al., 2009; Vaccaro et al., 2016; van der Bilt et al., 2010; van der Bilt et al., 2012; Wada et al., 2017; Weijenberg et al., 2013; Woda et al., 2010), as was the reliability of the method for assessing masticatory performance (*n* = 22; Felicio et al., 2008; Endo et al., 2014; Escudeiro Santos et al., 2006; Hama et al., 2014a; Hama et al., 2014b; Huggare, 1997; Kamiyama et al., 2010; Kapur, Yurkstas, & Soman, 1964; Khoury‐Ribas et al., 2017; Liedberg & Owall, 1995; Lujan‐Climent et al., 2008; Mahmood et al., 1992; Nokubi et al., 2013; Ohara et al., 2003; Sanchez‐Ayala et al., 2014; Sanchez‐Ayala et al., 2016; Sato et al., 2003; Schimmel et al., 2007; Schimmel et al., 2015; Silva, Nogueira, Rios, Schimmel, & Leles, 2018; van der Bilt et al., 2012; Weijenberg et al., 2013). Less frequently reported was measurement error (*n* = 9; Goto et al., 2016; Halazonetis et al., 2013; Lujan‐Climent et al., 2008; Matsui et al., 1996; Prinz, 1999; Sanchez‐Ayala et al., 2016; Schimmel et al., 2015; Silva et al., 2018; Sugiura et al., 2009) and responsiveness (*n* = 4; Asakawa et al., 2005; Fauzza & Lyons, 2008; Ishikawa et al., 2007; Mahmood et al., 1992). Finally, criterion validity (*n* = 6; Eberhard et al., 2012; Eberhard et al., 2015; Hama et al., 2014b; Kamiyama et al., 2010; Kobayashi et al., 2006; Sanchez‐Ayala et al., 2016) was presented, where four studies used comminution, and sieve as the gold standard (Eberhard et al., 2012; Eberhard et al., 2015; Kobayashi et al., 2006; Sanchez‐Ayala et al., 2016), and two (Hama et al., 2014b; Kamiyama et al., 2010) mixing ability methods adopted colorimeter values as the gold standard when assessing color mixture (Table 2).

### 3.2 Measurement properties of methods for assessing masticatory performance

Methods for assessing masticatory performance may be categorized into three main categories; that is, comminution methods (*n* = 21; Felicio et al., 2008; Eberhard et al., 2012; Eberhard et al., 2015; Escudeiro Santos et al., 2006; Fauzza & Lyons, 2008; Gunne, 1985; Huggare, 1997; Ikebe et al., 2005; Kapur et al., 1964; Khoury‐Ribas et al., 2017; Kobayashi et al., 2006; Lujan‐Climent et al., 2008; Mahmood et al., 1992; Mowlana et al., 1994; Nokubi et al., 2013; Ohara et al., 2003; Sanchez‐Ayala et al., 2014; Sanchez‐Ayala et al., 2016; Shiga et al., 2006; Slagter et al., 1993; Woda et al., 2010), mixing ability methods (*n* = 23; Abe et al., 2011; Asakawa et al., 2005; Endo et al., 2014; Halazonetis et al., 2013; Hama et al., 2014a; Hama et al., 2014b; Hayakawa et al., 1998; Ishikawa et al., 2007; Kamiyama et al., 2010; Liedberg & Owall, 1995; Matsui et al., 1996; Prinz, 1999; Sato et al., 2003; Schimmel et al., 2007; Schimmel et al., 2015; Silva et al., 2018; Speksnijder et al., 2009; Sugiura et al., 2009; Vaccaro et al., 2016; van der Bilt et al., 2010; van der Bilt et al., 2012; Wada et al., 2017; Weijenberg et al., 2013), or other methods (*n* = 2; Goto et al., 2016; Ikebe et al., 2010).

Only studies with methodological quality rated as fair, good, or excellent are reported in the results section. Studies rated as poor are described in Table S1.

### 3.3 Comminution methods

Comminution methods include all methods during which test food is comminuted into smaller particles, and particle sizes/volumes are assessed. Smaller particle sizes would indicate a better masticatory performance.

Definitions: Comminution methods fall into four categories:

- Sieve or optical scanning methods that assess fragmentation and particle‐size distribution with either single or multiple sieves or through some type of optical scanning and digital image analysis.
- Gummy jelly (GJ) methods that involve measuring glucose extraction released from chewed GJ; amount of released glucose is associated with the degree to which test food is fragmented and hence to masticatory performance.
- Fuchsin beads methods that use encapsulated fuchsin beads as test food to assess masticatory performance; fuchsin dye is release into the capsule when the beads are chewed, and the concentration of released dye, which is proportional to masticatory performance, is quantified with a spectrophotometer.
- Colorimetric methods that assess test food fragmentation through release or binding of dye from a solution; dye concentration is assessed with a spectrophotometer, which is proportional to masticatory performance.

### 3.4 Sieve and optical scanning methods

Forthcoming sections report measurement properties ratings in square brackets like this: [positive rating], [negative rating], [indeterminate rating].

One fair‐quality study (Mahmood et al., 1992) evaluated construct validity of optical scanning and use of image analysis to analyze fragmented test food particles [negative rating]. No studies of fair, good, or excellent quality reported on responsiveness.

Two good‐quality studies reported reliability of Optosil Comfort® (silicone material) as test food to assess masticatory performance using the sieve method [both positive rating] (Sanchez‐Ayala et al., 2014; Sanchez‐Ayala et al., 2016). Similarly, reliability of using alginate as a test food with the sieve method was reported in a fair‐quality study [negative rating] (Ohara et al., 2003). Another fair‐quality study reported reliability of a method using carrots as test food and analyzing particle size with a single sieve [positive rating] (Kapur et al., 1964). One fair‐quality study reported measurement error; the study used Optosil Comfort® (silicone) as a test material [indeterminate rating] (Sanchez‐Ayala et al., 2016).

### 3.5 Gummy jelly

Two fair‐ and good‐quality studies evaluated construct validity of a GJ as a test food. Both studies assessed masticatory performance using a glucose meter (Ikebe et al., 2005) or visual scale (Nokubi et al., 2013), respectively [both positive rating].

One good‐quality study reported reliability of a visual scale that was used with a GJ as test food [positive rating] (Nokubi et al., 2013).

No studies reported on responsiveness or measurement error.

### 3.6 Fuchsin beads

No studies of fair, good, or excellent quality reported on validity. One good‐quality study (Sanchez‐Ayala et al., 2016) reported reliability [negative rating]. No studies reported on responsiveness or measurement error.

### 3.7 Colorimetric methods

No studies of fair, good, or excellent quality reported on any of the measurement properties.

### 3.8 Mixing ability methods

For assessing masticatory performance, mixing ability methods involve two‐color gum or wax (as test food) and color‐changeable gum. The included studies described assessment of various digital analysis software apps and subjective color or bolus scales (Table S1).

### 3.9 Two‐color gum

An excellent‐quality study reported construct validity regarding two‐color gum Using MathLab 2015b, [positive rating] (Vaccaro et al., 2016). Two fair‐quality studies reported construct validity regarding use of ViewGum© for assessing masticatory performance with various types of two‐color gums [positive rating] (Halazonetis et al., 2013; Schimmel et al., 2015). One study of fair methodological quality using Adobe Photoshop CS2 reported conflicting research findings based on the age of the study participants, that is, negative findings were noted for young participants and positive findings for the elderly participants [indeterminate rating] (van der Bilt et al., 2010).

Several studies have attempted to establish the reliability of visual color or bolus scales that are used to assess masticatory performance with two‐color gums. One good‐quality study (Silva et al., 2018) reported that a two‐color gum visual scale enables reliable masticatory performance assessment as per visual and electronic colorimetric analyses [positive rating]. One fair‐quality study (Schimmel et al., 2015) assessed the same visual scale [positive rating]. A fair‐quality study (Endo et al., 2014) assessed another bolus and color scale and reported reliability [negative rating]. Measurement error was reported in three studies of fair (Halazonetis et al., 2013; Schimmel et al., 2015) and good (Silva et al., 2018) quality [all indeterminate rating].

### 3.10 Two‐color wax

One fair‐quality study (Sugiura et al., 2009) and one good‐quality study (Ikebe et al., 2010) reported construct validity when a two‐color wax was used in combination with a mixing ability test (Sugiura et al., 2009) /index (Ikebe et al., 2010) [positive rating for both]. One good‐quality study (Speksnijder et al., 2009) reported construct validity; the study involved another variant of a two‐color wax and a completely different mixing ability test; here, scanned wax was analyzed with Adobe Photoshop CS3 [positive rating]. Finally, a good‐quality study reported construct validity after comparing a subjective rating scale with a mixing ability method [positive rating] (van der Bilt et al., 2012). One fair‐quality study (Asakawa et al., 2005) reported responsiveness of a two‐color wax (Asakawa et al., 2005) after patients' masticatory performance was assessed before and after new dentures treatments [negative rating].

Two studies assessed reliability of two‐color wax. One poor‐quality study evaluated a mixing ability index and reported positive results for reliability [positive rating] (Sato et al., 2003). Another fair‐quality study assessed another mixing ability index and reported indeterminate reliability [indeterminate rating] (van der Bilt et al., 2012). A good quality study reported measurement error [indeterminate rating] (Sugiura et al., 2009).

### 3.11 Color‐changing gum

A color‐changing gum named Xylitol Masticatory Performance Evaluating Gum was used in several studies. Four fair‐quality studies reported the construct validity of this gum (Hama et al., 2014a; Wada et al., 2017), and two different color scales used in conjunction with the gum (Hama et al., 2014b; Kamiyama et al., 2010) [positive rating for all].

Reliability was reported in two studies using the same gum. Both methods rate the color change of the gum using two different color scales [positive rating] (Hama et al., 2014b; Kamiyama et al., 2010). Both studies were of fair‐quality studies. No studies of fair, good, or excellent quality reported on measurement error.

### 3.12 Other methods

One fair‐quality study (Ikebe et al., 2010) reported construct validity of the Eichner index, which measures the number of posterior occlusal contacts in relation to masticatory performance [positive rating].

### 3.13 Best evidence synthesis

The level of evidence is based on combining the studies' methodological quality and measurement properties rating (Table 3).

**Table 3:** Level of evidence

Two‐colored chewing gum							
	Study, first author	Method	Validity (criterion validity)	Validity (hypothesis testing)	Responsiveness	Reliability	Measurement error
	Schimmel et al. (2015)	Assessment of color mixing with digital software, ViewGum, on scanned wafer		Fair/Positive			
	Halazonetis et al. (2013)	Assessment of color mixing with digital software, ViewGum, on scanned wafer.		Fair/Positive			Fair/Indeterminate
Level of evidence				Moderate			Unknown
	Schimmel et al. (2007)	Assessment of color mixing with digital software, Adobe Photoshop Elements®, on scanned wafer		Poor/Positive			
	Endo et al. (2014)	Assessment of color mixing with digital software, Adobe Photoshop CS3®, on scanned wafer				Poor/indeterminate	Fair/Indeterminate
Level of evidence				Unknown		Unknown	Unknown
	van der Bilt et al. (2010)	Assessment of color mixing with digital software, Adobe Photoshop CS2®, on scanned wafer.		Fair/Negative for young test groupFair/Positive for elderly test group			
Level of evidence				Conflicting			
	Weijenberg et al. (2013)	Assessment of color mixing with a digital software, Mathematica, on photographed wafer.		Poor/Negative		Poor/Positive	
Level of evidence				Unknown		Unknown	
	Vaccaro et al. (2016)	Assessment of color mixing with a digital software, MATLAB 2015b, on photographed wafer		Excellent/Positive			
Level of evidence				Strong			
	Prinz (1999)	Assessment of two‐color chewing gum with digital image processing using Graphics Unbiased Measurement System.		Poor/indeterminate			Poor/indeterminate
Level of evidence				Unknown			Unknown
	Endo et al. (2014)	Subjective assessment of color mixing and bolus shaping with color scale 1–5, and Bolus Scale 1–4.				Fair/Negative	Fair/Indeterminate
	Liedberg and Owall (1995)					Poor/Indeterminate	
Level of evidence						Limited	Unknown
	Schimmel et al. (2007)	Subjective assessment of gum bolus, Scores 1–5 and assessment of color mixing of flattened gum wafer.				Poor/Negative	
	Schimmel et al. (2015)	Subjective assessment of gum bolus. Scores 1–5 and assessment of color mixing of flattened gum wafer.				Fair/Positive	Fair/Indeterminate
	Silvia et al. (2018)	Subjective assessment of color mixing. Scores 1–5.				Good/Positive	Good/Indeterminate
Level of evidence						Moderate	Unknown
Two‐colored wax							
	Study, first author	Method	Validity (criterion validity)	Validity (hypothesis testing)	Responsiveness	Reliability	Measurement error
	Asakawa et al. (2005)	Is a two colored wax and the Mixing Ability Index (MAI) able to detect difference of masticatory function before and after denture treatment?			Fair/Negative		
	Sato et al. (2003)	Assessment of color mixing and bolus shape and MP evaluated with the MAI.		Fair/Positive		Poor/Positive	
	Sugiura et al. (2009)	Assessment of color mixing and bolus shape and MP evaluated with the MAI.		Good/Positive			Good/Indeterminate
Level of evidence				Moderate	Limited	Unknown	Unknown
	Speksnijder et al. (2009)	MP assessed with two‐colored wax and Mixing Ability Test that assesses color mixing. Assessment of color mixing with digital image software, Adobe Photoshop CS3.		Good/Positive			
Level of evidence				Moderate			
	van der Bilt et al. (2012)	MP assessed with two‐colored wax and Mixing Ability Test (MAT). Evaluation of visual assessment of MAT compared with a digital image analysis with Adobe Photoshop CS3.		Good/Positive		Good/Indeterminate	
Level of evidence				Moderate		Unknown	
Color changeable chewing gum							
	Study, first author	Method	Validity (criterion validity)	Validity (hypothesis testing)	Responsiveness	Reliability	Measurement error
	Hama et al. (2014a)	Assessment of MP w/Masticatory Performance Evaluating Gum XYLITOL. Color scale, score 1–11, to assess color change in gum.	Fair/Positive			Fair/Positive	
	Hama et al. (2014b)	Assessment of color change inMasticatory Performance Evaluating Gum XYLITOL to rate MP.		Poor/Positive		Poor/Positive	
	Wada et al. (2017)	Masticatory Performance Evaluating Gum XYLITOL		Fair/Positive			
	Ishikawa et al. (2007)	Color change of Masticatory Performance Evaluating Gum XYLITOLColor change assessed with colorimeter.			Poor/Indeterminate		
Level of evidence			Limited	Limited	Unknown	Limited	
	Kamiyama et al. (2010)	Assessment of color scale used to rate color change of Masticatory Performance Evaluating Gum XYLITOL.	Fair/positive			Fair/Positive	
Level of evidence			Limited			Limited	
	Hayakawa et al. (1998)	Assessment of color changeable chewing gum and color scale for assessment of MP.		Poor/Positive			
Level of evidence				Unknown			
	Matsui et al. (1996)	Evaluation of color changeable chewing gum ax a test food to assess MP.		Poor/Indeterminate			Poor/Indeterminate
Level of evidence				Unknown			Unknown
Mixing & video endoscopic tests							
	Abe et al. 2011	Two‐colored rice and uirou (rice cake)mixing assessed with video endoscopic in the oropharynx		Poor/Positive			
Level of evidence				Unknown			
Sieve & optical scanning tests							
	Study, first author	Method	Validity (criterion validity)	Validity (hypothesis testing)	Responsiveness	Reliability	Measurement error
	Eberhard et al. (2012)	Assessment of optical scanning as a method to analyze fragmented test food particles. Flatbed scanner and ImageJ software and Xnview software.	Poor/Positive				
	Eberhard et al. (2015)	Assessment of optical scanning as a method to analyze fragmented test food particles of denture wearers. Flatbed scanner and ImageJ and Xnview software.	Poor/Positive				
Level of evidence			Unknown				
	Mowlana et al. (1994)	Assessment of optical scanning as a method to analyze fragmented test food particles, using flatbed scanner and digital image software.		Poor/Indeterminate			
Level of evidence				Unknown			
	Mahmood et al. (1992)			Fair/Negative	Poor/Negative	Poor/Indeterminate	
Level of evidence				Limited	Unknown	Unknown	
	Slagter et al. (1993)	Comparison of Optocal and Optosil as a test food with sieve test.		Poor/Positive			
Level of evidence				Unknown			
	Sanchez‐Ayala et al. 2014	Assessment of Optosil Comfort®as an artificial test food for MP evaluation using sieve method.				Good/Positive	
	Sanchez‐Ayala et al. (2016)	Assessment of Optosil Comfort® as an artificial test food. For MP evaluation using sieve method.				Good/Positive	Fair/Indeterminate
Level of evidence						Strong	Unknown
	Khoury‐Ribas et al. (2017)	Assessment of Optosil Plus® and sieve as a method to assess MP.				Poor/Negative	
	Lujan‐Climent et al. (2008)	Assessment of Optosil Plus® and sieve as a method to assess MP.				Poor/Positive	Poor/Indeterminate
Level of evidence						Unknown	Unknown
	Fauzza & Lyons (2008)	Assessment of alginate as a test food to assess MP in denture wearers.			Poor/Indeterminate		
	Ohara et al. (2003)	Assessment of alginate as a test food and sieve method to assess MP.		Poor/Positive		Fair/Negative	
Level of evidence				Unknown	Unknown	Limited	
	Khoury‐Ribas et al. (2017)	Assessment of Optozeta® and sieve as a method to assess MP.		Poor/Positive		Poor/Negative	
Level of evidence				Unknown		Unknown	
	Woda et al. (2010)	Assessment of MP with masticatory normative indicator.		Poor/Positive			
Level of evidence				Unknown			
	Kapur et al. (1964)	Valuation of carrot as test food to evaluate MP in dentures wearers. Single sieve.				Fair/Positive	
Level of evidence						Limited	
Gummy jelly							
	Study, first author	Method	Validity (criterion validity)	Validity (hypothesis testing)	Responsiveness	Reliability	Measurement error
	Nokubi et al. (2013)	Assessment of a visual scoring scale, 1–10,to assess MP with gummy jelly as test food.		Fair/Positive		Good/Positive	
	Ikebe et al. (2005)	Evaluation of gummy jellyas a test food to assess MP.		Poor/Positive			
Level of evidence				Limited		Moderate	
	Kobayashi et al. (2006)	Evaluation of gummy jelly as a test food to assess MP.	Poor/Positive				
Level of evidence			Unknown				
	Shiga et al. (2006)	Evaluation of a blood glucose meter to assess MP w/gummy jelly.		Poor/Positive			
Level of evidence				Limited			
Fuchsin beads							
	Study, first author	Method	Validity (criterion validity)	Validity (hypothesis testing)	Responsiveness	Reliability	Measurement error
	Escudeiro Santos (2006)	Evaluation of encapsulated fuchsine beadsas a method to assess MP				Poor/indeterminate	
	Felicio et al. (2008)	Evaluation of encapsulated fuchsine beads as a method to assess MP.		Poor/Positive		Poor/indeterminate	
	Sanchez‐Ayala et al. (2016)	Evaluation of encapsulated fuchsine beads as a method to assess MP.	Good/Negative			Good/Indeterminate	
Level of evidence			Moderate	Unknown		Unknown	
Colormetric tests							
	Study, first author	Method	Validity (criterion validity)	Validity (hypothesis testing)	Responsiveness	Reliability	Measurement error
	Huggare (1997)	Evaluation of color bindingtablets to assess MP.		Poor/Positive		Poor/Indeterminate	
Level of evidence				Unknown		Unknown	
	Gunne (1985)	Evaluation of gelatin hardened by formalin as test food to assess MP.		Poor/Indeterminate			
Level of evidence				Unknown			
Other methods							
	Study, first author	Method	Validity (criterion validity)	Validity (hypothesis testing)	Responsiveness	Reliability	Measurement error
	Ikebe et al. (2010)	Assessment of MP with Eichner index.		Fair/Positive			
Level of evidence				Limited			
	Goto et al. (2016)	Assessment of MP with an odor sensor device, OMX‐SR		Poor/Positive			Poor/Indeterminate
Level of evidence				Unknown			Unknown

*Note*. MP: masticatory performance; NR: not reported; CS: chewing strokes; SD: standard deviation.

#### 3.13.1 Comminution methods

### 3.14 Sieve and optical scanning methods

Limited or unknown level of evidence was reported for criterion validity (Eberhard et al., 2012; Eberhard et al., 2015) and construct validity (Mahmood et al., 1992; Mowlana et al., 1994; Ohara et al., 2003; Slagter et al., 1993). Unknown level of evidence was also reported for a universal indicator to differentiate normal and impaired masticatory performance (Woda et al., 2010). Two studies reported unknown level of evidence for responsiveness (Fauzza & Lyons, 2008; Mahmood et al., 1992). Optosil Comfort® (silicone material) as test food with the sieve method reported strong level of evidence for reliability in two studies (Sanchez‐Ayala et al., 2014; Sanchez‐Ayala et al., 2016). All other studies reported limited or no level of evidence for reliability (Fauzza & Lyons, 2008; Kapur et al., 1964; Khoury‐Ribas et al., 2017; Lujan‐Climent et al., 2008; Mahmood et al., 1992; Ohara et al., 2003) while two studies reported unknown level of evidence for measurement error (Lujan‐Climent et al., 2008; Sanchez‐Ayala et al., 2016).

### 3.15 GJ methods

Limited/unknown level of evidence was reported for criterion validity (Kobayashi et al., 2006) or construct validity (Ikebe et al., 2005; Nokubi et al., 2013). Unknown level of evidence was reported regarding construct validity when using a glucose meter (Shiga et al., 2006). Moderate level of evidence was reported regarding reliability of a 1 to 10‐point visual scale that was used with GJ test food (Nokubi et al., 2013),

### 3.16 Fuchsin beads method

Moderate level of evidence was reported for criterion validity (Sanchez‐Ayala et al., 2016) and unknown evidence for construct validity (Felicio et al., 2008). Unknown level of evidence reliability was reported in three studies (Felicio et al., 2008; Escudeiro Santos et al., 2006; Sanchez‐Ayala et al., 2016).

### 3.17 Colorimetric methods

Two studies reported unknown level of evidence regarding construct validity (Gunne, 1985; Huggare, 1997). One study reported unknown level of evidence for reliability (Huggare, 1997).

#### 3.17.1 Mixing methods

Strong level of evidence was reported for construct validity when using (a) two‐color (red‐white) gum and (b) MatLab 2015b to analyze variance of hue histograms on a young population (age: females, 25 ± 5.8; males, 25 ± 6.8). Moderate level of evidence was reported for construct validity when using (a) two‐color gum with azure–pink, blue–red, or green–dark violet and (b) ViewGum® (image software) to analyze standard deviation/variance of hue in dentate groups, edentulous groups, and in persons with overdentures (Halazonetis et al., 2013; Schimmel et al., 2015). All other studies reported unknown, limited, or conflicting level of evidence for construct validity of two‐color gum methods (Endo et al., 2014; Schimmel et al., 2007; van der Bilt et al., 2010; Weijenberg et al., 2013).

Two studies reported moderate level of evidence for construct validity when using two‐color wax and a mixing ability index, to assess masticatory performance in fully dentate or partially edentulous (Sato et al., 2003; Sugiura et al., 2009). Moderate level of evidence for construct validity was also reported in one study that used a two‐color, blue–red wax, and digital image software to analyze the standard of intensity of distribution (Speksnijder et al., 2009) in dentate or in persons with dentures or overdentures or full dentures. Yet, another study reported moderate level of evidence for construct validity regarding two‐color wax (van der Bilt et al., 2012).

Limited/unknown level of evidence was reported for criterion validity (Hama et al., 2014b; Kamiyama et al., 2010) and construct validity (Hayakawa et al., 1998; Matsui et al., 1996; Prinz, 1999) for color‐changeable gums used as test food.

Unknown level of evidence was reported for assessment of a two‐color mixture of a food bolus using videoendoscopy (Abe et al., 2011).

Only three studies reported limited/unknown level of evidence for all mixing ability methods (Asakawa et al., 2005; Ishikawa et al., 2007; Wada et al., 2017).

Moderate level of evidence was reported for reliability of a visual color scale and a bolus scale used to assess mixing ability and masticatory performance (Silva et al., 2018). Limited/unknown level of evidence was reported for all other types of mixing ability methods, regardless of whether the method involved optical scanning/photography and digital image analysis or subjective assessment using visual scales (Endo et al., 2014; Hama et al., 2014b; Kamiyama et al., 2010; Liedberg & Owall, 1995; Sato et al., 2003; Schimmel et al., 2007; Schimmel et al., 2015; van der Bilt et al., 2012; Weijenberg et al., 2013). Seven studies reported unknown level of evidence for measurement error (Endo et al., 2014; Halazonetis et al., 2013; Matsui et al., 1996; Prinz, 1999; Schimmel et al., 2015; Speksnijder et al., 2009; Sugiura et al., 2009).

#### 3.17.2 Other methods

### 3.18 Eichner index and odor sensor device

Limited/unknown level of evidence was reported for construct validity regarding two different methods for assessing masticatory performance: Eichner index (Ikebe et al., 2010) and an odor sensor device (Goto et al., 2016). Unknown level of evidence was also reported for measurement error for the odor sensor device (Goto et al., 2016).

To summarize, the studies reporting methods using two‐color chewing gums and digital analysis revealed moderate to strong level of evidence for construct validity (Halazonetis et al., 2013; Schimmel et al., 2015; Vaccaro et al., 2016), and moderate level of evidence for reliability using a visual scale (Silva et al., 2018). Moderate level of evidence was also reported for construct validity using two‐colored wax (Speksnijder et al., 2009; Sugiura et al., 2009; van der Bilt et al., 2012). Strong level of evidence was reported for reliability using Optosil Comfort as a test food with multiple sieve method (Sanchez‐Ayala et al., 2014; Sanchez‐Ayala et al., 2016). Finally, moderate level of evidence was reported for reliability using GJ as a test food and using a visual scale for assessment (Nokubi et al., 2013).

## 4 DISCUSSION

The present systematic review investigated 46 studies that reported measurement properties of methods for assessing masticatory performance. These studies accounted for persons ages ≥18, with varying dentitions and tooth replacements. No study reported findings associated with all four measurement properties. The present systematic review found that for:

- Construct validity, moderate‐to‐strong levels of evidence were reported for two‐color gum or wax via digital software analyses. Limited level of evidence was reported regarding comminution, GJ, and fuchsine beads.
- Reliability, moderate level of evidence was reported regarding a visual scale in a clinical setting with two‐color chewing gum as test food. Moderate‐to‐strong level of evidence was reported for (a) silicone cubes and particle analysis with sieves for the comminution method and (b) a visual scale with the GJ.

Three reviews have addressed masticatory efficiency, performance, and function (Boretti, Bickel, & Geering, 1995; Oliveira et al., 2014; Tarkowska, Katzer, & Ahlers, 2017). However, these reviews have not attempted to identify specifically studies that use methods for objectively assessing masticatory performance or evaluated the measurement properties of methods for assessing masticatory performance with a validated appraisal tool such as COSMIN.

Our findings corroborate the conclusion in one of these reviews, where that a two‐color chewing gum method is valid and reliable and can be used in different populations (Tarkowska et al., 2017). However, one of the other reviews considered the comminution/sieve method to be the gold standard when assessing masticatory performance in denture wearers (Oliveira et al., 2014). Finally, one older review from 1995 emphasized a sociopsychologic approach than a biomedical. Thus, assessment of patients subjective masticatory ability is stressed in contrast to masticatory performance, especially for patients using dentures (Boretti et al., 1995). Studies have shown a weak correlation between masticatory performance and subjective masticatory ability (Pedroni‐Pereira et al., 2018; Slagter, Olthoff, Bosnian, & Steen, 1992; van der Bilt, Olthoff, Bosman, & Oosterhaven, 1994). This systematic review increases the knowledge regarding the validity and reliability as included studies have been evaluated using accurate tools as COSMIN, and findings have been summarized using a standardized method that previous reviews have not provided.

Two main methods for assessing masticatory performance can be identified.

4.1

#### 4.1.1. Comminution method

Two studies (Sanchez‐Ayala et al., 2014; Sanchez‐Ayala et al., 2016) reported strong level of evidence for reliability when using Optosil Comfort® as a test food with the sieve method. This method requires resources, such as lab equipment, takes a lot of time, and is probably best suited for research.

Moderate level of evidence was reported for reliability of a 1‐ to 10‐point visual scale (used with GJ test food; Nokubi et al., 2013). This method seems to be best suited for clinical settings.

One study compared two foods and methods (a) fuchsine beads and ultraviolet–visible spectrophotometry and (b) silicone cubes and multiple sieving as the gold standard (Sanchez‐Ayala et al., 2016). The study reported moderate but negative level of evidence for criterion validity in a younger study population where the sieve method, with Optosil Comfort® as test food, was used as gold standard. Here also, the methods require lab equipment.

#### 4.1.2. Mixing ability method

Regarding construct validity, six studies reported moderate level of evidence (Halazonetis et al., 2013; Sato et al., 2003; Schimmel et al., 2015; Speksnijder et al., 2009; Sugiura et al., 2009; van der Bilt et al., 2012), and one study reported strong level of evidence (Vaccaro et al., 2016). Regarding reliability, moderate level of evidence was reported in one study that used a visual bolus/color scale (Silva et al., 2018).

There seem to be evidence for construct validity and reliability for two‐color gum and wax used in populations with (a) complete or compromised dentitions and (b) complete or implant‐supported dentures. That said, the method mostly requires optical and image processing. A visual bolus/color scale is probably useful in a clinical setting.

The next section addresses measurement properties of methods for assessing masticatory performance.

#### 4.1.3. Measurement properties

The studies reported two types of validity: construct and criterion validity.

Construct validity is often tested with predefined hypotheses, but many studies reported vague or no specific hypotheses. Hypotheses often formulate the relationship of the scores of the instrument, compared with scores of other instruments that measure similar or dissimilar constructs (convergent and discriminant validity) or to differences between subgroups of patients. Similar constructs, in this case, often included bite force, other methods for assessing masticatory performance, electromyography activity, and chewing cycles. The studies categorized participants into age groups, dentitions groups, and or prosthetic treatment groups.

Hypotheses should state magnitude and direction of measurement scores, and this is a problem, because no quantifiable criteria or defined distinction exists that would allow to discriminate between different functional levels of masticatory performance. That said, efforts to develop such a universal indicator (Woda et al., 2010) occurred. The following questions are raised: What food particle size or color mixture should a masticatory performance test be able to discriminate? What magnitude of difference would be clinically relevant (i.e., minimal important changes) for patients? What is necessary for a method to be considered better than another?

Some methods were assessed for criterion validity, namely, the degree to which the score of the tested instrument correlates with a golden standard that measures the same construct. Studies that evaluated criterion validity used the comminution and sieve method as a gold standard (Eberhard et al., 2012; Eberhard et al., 2015; Kobayashi et al., 2006; Sanchez‐Ayala et al., 2016) or a colorimeter when assessing color mixture (Hama et al., 2014b; Kamiyama et al., 2010). But criterion validity could be questionable because comminution and mixing ability methods may not measure the same masticatory performance characteristics of the masticatory performance process.

Only four studies reported on responsiveness. These provided limited/unknown level of evidence because of low sample size (Fauzza & Lyons, 2008; Ishikawa et al., 2007; Mahmood et al., 1992), vaguely formulated hypotheses (Asakawa et al., 2005), and insufficient clarity regarding whether or not, a change occurred among the study participants (Asakawa et al., 2005; Fauzza & Lyons, 2008), Level of evidence for responsiveness is a problem because need for adequate methods exists for assessing effects of interventions for enhancing masticatory performance, particularly in the aging population. Studies have revealed possible association between good nutritional status and oral health regarding dental condition in the elderly (Van Lancker et al., 2012).

##### Reliability and measurement error

Reliability indicates the degree to which an instrument can distinguish patients from each other, while measurement error addresses magnitude of measurement error (HCWd et al., 2015). Reliability is an important factor if the instrument is to distinguish between poor, mediocre, and good masticatory performance, while quantification of measurement error is important to discern if a change in score is real or caused by measurement error (de Vet, Terwee, Knol, & Bouter, 2006; HCWd et al., 2015). Although measurement error is an important parameter for assessments, it is clear from this review that reliability is the preferred measurement property to assess. Five studies assessed measurement error but none defined minimal important changes or smallest detectable change. Measurement error can be derived from the intraclass correlation coefficient formula, but this was usually not reported.

## 5 METHODOLOGICAL CONSIDERATIONS

The publication period of the included studies ranged from 1964 to 2018. Articles published during the latter third of this period, especially during 2010–2018, tended to report study design and methodology (e.g., choice of included statistical models) in a more explicit way and more in accordance to the COSMIN standards. Hence, these studies were generally rated with higher methodological scores. Traditional methods generally received lower ratings for methodological quality (e.g., comminution/sieve methods); because, measurement properties were assessed in studies published during the earlier part of this period. It is possible that comminution methods would be rated higher if the methodology would have been more explicitly describe, as they usually are in studies published the last 10 years.

COSMIN was originally designed to assess measurement properties of health‐related and patient‐reported outcomes and has been used in other systematic reviews to evaluate diagnostic tests and methods to establish performance (Dunaway Young et al., 2016; Kroman, Roos, Bennell, Hinman, & Dobson, 2014). COSMIN was therefore considered relevant for assessing the measurement properties of methods for assessing masticatory performance.

In the included studies, sample size had to be considered because power calculation or confidence interval data were lacking and could indicate statistical precision. COSMIN requires a sample size of *n* ≥ 30 for a fair, and *n* ≥ 50 (Terwee et al., 2012) for good grade of methodological quality. In addition, two‐thirds of the studies had low sample sizes, and the methods varied too much in their mechanics or study populations to pool studies that assessed similar methods. Because the COSMIN guidelines were originally created to evaluate questionnaires, the sample size requirements do not necessarily apply to studies reporting on performance‐based measures. Here, smaller sample sizes may produce a large enough effect size, but this review followed COSMIN requirements.

Many studies could probably be regarded as pilot studies, even if continuing main studies could not be found. Some methods require lab‐intensive equipment, such as sieves or digital image software (Halazonetis et al., 2013; Sanchez‐Ayala et al., 2014; Sanchez‐Ayala et al., 2016; Sato et al., 2003; Schimmel et al., 2015; Speksnijder et al., 2009; Sugiura et al., 2009; Vaccaro et al., 2016), and only a few methods suite a clinical setting (Nokubi et al., 2013; Silva et al., 2018). Concerning generalizability, the level of evidence for measurement properties is only generalizable to populations with similar characteristics as the study population.

Studies not published in full text or English were excluded; consequently, additional information on measurement properties and descriptions of methods for assessing masticatory performance might have been missed that potentially may have affected the level of evidence.

## 6 CONCLUSIONS

In a clinical setting or as a diagnostic test, there is no established method for assessing masticatory performance with a strong level of evidence for all measurement properties. All available assessment methods with variable level of evidence require lab‐intensive equipment, such as sieves or digital image software. Clinical trials with sufficient sample size, to infer trueness and precision, are needed for evaluating diagnostic values of available methods for assessing masticatory performance.

## CONFLICT OF INTERESTS

The authors have nothing to disclose.

## Supporting information

Data S1
Supporting information

Data S2
Supporting information

Data S3
Supporting information

Table S1 Supporting information
