# Supplementary material for: Systematic review of measurement properties of methods for objectively assessing masticatory performance
Source: Clin Exp Dent Res. 2019 Jan 31;5(1):76–104. doi: 10.1002/cre2.154 (PMC6392827; doi:10.1002/cre2.154)
Supplement: Supplementary file 1 — Data S1 Supporting information [file CRE2-5-76-s001.docx]

Documentation of search strategies

University Library search consultation group

Date: January 2017

Topic/research question: Methods for objectively assessing clinical masticatory performance

Name of researcher(s): Per Stjernfeldt-Elgestad, Department of Dental Medicine

Librarian(s): Carl Gornitzki & Susanne Gustafsson

Databases:

1. Medline (Ovid)
2. Embase (embase.com)
3. Web of Science Core Collection
4. Cochrane Library (Wiley)
5. ...

Total number of hits:

- Before deduplication:
- After deduplication:

Comments:

**PRISMA 2009 Flow Diagram^[[1]](#footnote-1)^**

**Records identified through database searching
(n = )**

## Screening

## Included

## Eligibility

## Identification

Additional records identified through other sources
(n = )

**Records after duplicates removed
(n = )**

Records screened
(n = )

Records excluded
(n = )

Full-text articles assessed for eligibility
(n = )

Full-text articles excluded, with reasons
(n = )

Studies included in qualitative synthesis
(n = )

Studies included in quantitative synthesis (meta-analysis)
(n = )

1. Medline (Ovid)

| Date of Search: 2017-01-26  Number of hits: 6,124  Comments: | Field labels: |
| --- | --- |
| 1. Mastication/  2. Bite Force/  3. (masticat* or oral function or chewed or chewing or bite force or dentition* or denture*).ti,ab,kf.  4. or/1-3  5. Chewing Gum/  6. Deglutition/  7. exp Food/  8. Dental Stress Analysis/  9. (sieving or sieve or comminution or swallow* or saliva* or gum or jell* or wax or food).ti,ab,kf.  10. or/5-9  11. Trail Making Test/  12. Psychological tests/  13. Neuropsychological Tests/  14. Health status indicators/  15. Severity of illness index/  16. Karnofsky performance status/  17. Sickness impact profile/  18. Psychometrics/  19. "Reproducibility of Results"/  20. (measur* or assess* or evaluat* or test* or inventor* or battery or indicator* or profile or index or indices or scale* or instrument* or psychometric* or reproduc* or reliable or reliability or valid*).ti,ab,kf.  21. or/11-20  22. 4 and 10 and 21  23. limit 22 to english language  24. (animals not humans).sh.  25. 23 not 24  26. limit 25 to yr="1860 - 2000"  27. limit 25 to yr="2000 -Current"  28. remove duplicates from 26  29. remove duplicates from 27  30. 28 or 29 | |

2. Embase (embase.com)

| Date of Search: 2017-01-26  Number of hits: 4,746  Comments: | Field labels: |
| --- | --- |
| **#1** **'mastication'**/de  **#2** **masticat***:ti,ab OR **'oral function'**:ti,ab OR **chewed**:ti,ab OR **chewing**:ti,ab OR **'bite force'**:ti,ab OR **dentition***:ti,ab OR **denture***:ti,ab  **#3** **#1** OR **#2**  **#4** **'chewing gum'**/de  **#5** **'swallowing'**/de  **#6** **'food'**/exp  **#7** **sieving**:ti,ab OR **sieve**:ti,ab OR **comminution**:ti,ab OR **swallow***:ti,ab OR **saliva***:ti,ab OR **gum**:ti,ab OR **jell***:ti,ab OR **wax**:ti,ab OR **food**:ti,ab  **#8** **#4** OR **#5** OR **#6** OR **#7**  **#9** **'psychologic test'**/de  **#10** **'neuropsychological test'**/de  **#11** **'health status indicator'**/de  **#12** **'severity of illness index'**/de  **#13** **'karnofsky performance status'**/de  **#14** **'sickness impact profile'**/de  **#15** **'psychometry'**/de  **#16** **'reproducibility'**/de  **#17** **measur***:ti,ab OR **assess***:ti,ab OR **evaluat***:ti,ab OR **test***:ti,ab OR **inventor***:ti,ab OR **battery**:ti,ab OR **indicator***:ti,ab OR **profile**:ti,ab OR **index**:ti,ab OR **indices**:ti,ab OR **scale***:ti,ab OR **instrument***:ti,ab OR **psychometric***:ti,ab OR **reproduc***:ti,ab OR **reliable**:ti,ab OR **reliability**:ti,ab OR **valid***:ti,ab  **#18** **#9** OR **#10** OR **#11** OR **#12** OR **#13** OR **#14** OR **#15** OR **#16** OR **#17**  **#19** **#3** AND **#8** AND **#18**  **#20** **#3** AND **#8** AND **#18** AND [english]/lim  **#21** [animals]/lim NOT [humans]/lim  **#22** **#20** NOT **#21**  **#23** **#22** AND (**'article'**/it OR **'article in press'**/it OR **'conference paper'**/it OR **'review'**/it) | |

3. Web of Science Core Collection

| Date of Search: 2017-01-26  Number of hits: 5,539  Comments: | Field labels: |
| --- | --- |
| (TS=(masticat* OR "oral function" OR chewed OR chewing OR "bite force" OR dentition* OR denture*)  AND  TS=(sieving OR sieve OR comminution OR swallow* OR saliva* OR gum OR jell* OR wax OR food)  AND  TS=(measur* OR assess* OR evaluat* OR test* OR inventor* OR battery OR indicator* OR profile OR index OR indices OR scale* OR instrument* OR psychometric* OR reproduc* OR reliable OR reliability OR valid*))  *AND*  **LANGUAGE:** (English) | |

4. Cochrane Library (Wiley)

| Date of Search: 2017-01-26  Number of hits: 1,023   - Cochrane Reviews = 42 - Other Reviews = 4 - Trials = 976 - Economic Evaluations = 1   Comments: | Field labels: |
| --- | --- |
| ID Search Hits  #1 masticat* or "oral function" or chewed or chewing or "bite force" or dentition* or denture*:ti,ab,kw (Word variations have been searched) 3851  #2 sieving or sieve or comminution or swallow* or saliva* or gum or jell* or wax or food:ti,ab,kw (Word variations have been searched) 33428  #3 measur* or assess* or evaluat* or test* or inventor* or battery or indicator* or profile or index or indices or scale* or instrument* or psychometric* or reproduc* or reliable or reliability or valid*:ti,ab,kw (Word variations have been searched) 634127  #4 #1 and #2 and #3 1023 | |

5. Cinahl (Ebsco)

| Date of Search: 2017-01-26  Number of hits:  Comments: | Field labels: |
| --- | --- |
| \| **#** \| **Query** \| **Results** \| \| --- \| --- \| --- \| \| S1 \| (MH "Mastication") \| 987 \| \| S2 \| (MH "Bite Force") \| 396 \| \| S3 \| TI ( masticat* OR "oral function" OR chewed OR chewing OR "bite force" OR dentition* OR denture* ) OR AB ( masticat* OR "oral function" OR chewed OR chewing OR "bite force" OR dentition* OR denture* ) \| 5,248 \| \| S4 \| S1 OR S2 OR S3 \| 5,707 \| \| S5 \| (MH "Chewing Gum") \| 499 \| \| S6 \| (MH "Deglutition") \| 1,928 \| \| S7 \| (MH "Deglutition Disorders") \| 4,027 \| \| S8 \| (MH "Food+") \| 77,464 \| \| S9 \| TI ( sieving OR sieve OR comminution OR swallow* OR saliva* OR gum OR jell* OR wax OR food ) OR AB ( sieving OR sieve OR comminution OR swallow* OR saliva* OR gum OR jell* OR wax OR food ) \| 48,792 \| \| S10 \| S5 OR S6 OR S7 OR S8 OR S9 \| 116,002 \| \| S11 \| (MH "Psychological Tests") \| 44,713 \| \| S12 \| (MH "Neuropsychological Tests") \| 18,778 \| \| S13 \| (MH "Health Status Indicators") \| 7,099 \| \| S14 \| (MH "Severity of Illness Indices+") \| 25,430 \| \| S15 \| (MH "Karnofsky Performance Status") \| 1,020 \| \| S16 \| (MH "Sickness Impact Profile") \| 1,217 \| \| S17 \| (MH "Psychometrics") \| 9,313 \| \| S18 \| (MH "Reproducibility of Results") \| 16,820 \| \| S19 \| TI ( measur* OR assess* OR evaluat* OR test* OR inventor* OR battery OR indicator* OR profile OR index OR indices OR scale* OR instrument* OR psychometric* OR reproduc* OR reliable OR reliability OR valid* ) OR AB ( measur* OR assess* OR evaluat* OR test* OR inventor* OR battery OR indicator* OR profile OR index OR indices OR scale* OR instrument* OR psychometric* OR reproduc* OR reliable OR reliability OR valid* ) \| 833,081 \| \| S20 \| S11 OR S12 OR S13 OR S14 OR S15 OR S16 OR S17 OR S18 OR S19 \| 867,277 \| \| S21 \| S3 AND S10 AND S20 \| 737 \| \| S22 \| S3 AND S10 AND S20 \| 730 \| \| S23 \| S3 AND S10 AND S20 \| 622 \| \| S24 \| S3 AND S10 AND S20 \| 730 \| | |

1. From: Moher D, Liberati A, Tetzlaff J, Altman DG, The PRISMA Group (2009). Preferred Reporting Items for Systematic Reviews and Meta-Analyses: The PRISMA Statement. PLoS Med 6(6): e1000097. doi:10.1371/journal.pmed1000097. For more information, visit [www.prisma-statement.org](http://www.consort-statement.org/). [↑](#footnote-ref-1)
