# Supplementary material for: Systematic review of measurement properties of methods for objectively assessing masticatory performance
Source: Clin Exp Dent Res. 2019 Jan 31;5(1):76–104. doi: 10.1002/cre2.154 (PMC6392827; doi:10.1002/cre2.154)
Supplement: Supplementary file 2 — Data S2 Supporting information [file CRE2-5-76-s002.docx]

**Excluded full text articles n=36**

**^1-36^**

1. Al-Ali F, Heath MR, Wright PS. Simplified method of estimating masticatory performance. J Oral Rehabil. 1999;26(8):678-83.

2. Albert TE, Buschang PH, Throckmorton GS. Masticatory performance: a protocol for standardized production of an artificial test food. J Oral Rehabil. 2003;30(7):720-2.

3. Anastassiadou V, Heath MR. The development of a simple objective test of mastication suitable for older people, using chewing gums. Gerodontology. 2001;18(2):79-86.

4. Baragar FA, van der Bilt A, van der Glas HW. An analytic probability density for particle size in human mastication. J Theor Biol. 1996;181(2):169-78.

5. Campos SS, Pereira CV, Zangeronimo MG, Marques LS, Pereira LJ. Influence of disinfectant solutions on test materials used for the determination of masticatory performance. Pesqui Odontol Bras. 2013;27(3):238-44.

6. Cazal MS, da Silva AM, Galo R, Junior WM, da Silva MA. Comparison of dynamic electromyographic analysis of masticatory capsules with materials of different textures. Cranio. 2015:1-7.

7. Chong-Shan S, Guan O, Tian-Wen G. Masticatory efficiency determined with direct measurement of food particles masticated by subjects with natural dentitions. Journal of Prosthetic Dentistry.64(6):723-6.

8. Compagnon D, Veyrune JL, Morenas M, Faulks D. Development of a synthetic bolus using silicone elastomer for the study of masticatory efficiency. J Prosthet Dent. 1999;81(6):704-9.

9. Edlund J, Lamm CJ. Masticatory efficiency. J Oral Rehabil. 1980;7(2):123-30.

10. Gunne HS. Masticatory efficiency. A new method for determination of the breakdown of masticated test material. Acta Odontol Scand. 1983;41(5):271-6.

11. Hennequin M, Allison PJ, Veyrune JL, Faye M, Peyron M. Clinical evaluation of mastication: validation of video versus electromyography. Clin Nutr. 2005;24(2):314-20.

12. Kayser AF, van der Hoeven JS. Colorimetric determination of the masticatory performance. J Oral Rehabil. 1977;4(2):145-8.

13. Lucas PW, Luke DA. Methods for analysing the breakdown of food in human mastication. Arch Oral Biol. 1983;28(9):813-9.

14. Mowlana F, Heath R. Assessment of masticatory efficiency: new methods appropriate for clinical research in dental practice. Eur J Prosthodont Restor Dent. 1993;1(3):121-5.

15. Nakasima A, Higashi K, Ichinose M. A new, simple and accurate method for evaluating masticatory ability. J Oral Rehabil. 1989;16(4):373-80.

16. Nicolas E, Veyrune JL, Lassauzay C, Peyron MA, Hennequin M. Validation of video versus electromyography for chewing evaluation of the elderly wearing a complete denture. J Oral Rehabil. 2007;34(8):566-71.

17. Nokubi T, Nokubi F, Yoshimuta Y, Ikebe K, Ono T, Maeda Y. Measuring masticatory performance using a new device and beta-carotene in test gummy jelly. J Oral Rehabil. 2010;37(11):820-6.

18. Nokubi T, Yasui S, Yoshimuta Y, Kida M, Kusunoki C, Ono T, et al. Fully automatic measuring system for assessing masticatory performance using beta-carotene-containing gummy jelly. J Oral Rehabil. 2013;40(2):99-105.

19. Pocztaruk Rde L, Frasca LC, Rivaldo EG, Fernandes Ede L, Gaviao MB. Protocol for production of a chewable material for masticatory function tests (Optocal - Brazilian version). Pesqui Odontol Bras. 2008;22(4):305-10.

20. Prinz JF. Quantitative evaluation of the effect of bolus size and number of chewing strokes on the intra-oral mixing of a two-colour chewing gum. J Oral Rehabil. 1999;26(3):243-7.

21. Salleh NM, Fueki K, Garrett NR, Ohyama T. Objective and subjective hardness of a test item used for evaluating food mixing ability. J Oral Rehabil. 2007;34(3):174-83.

22. Sato H, Fueki K, Sueda S, Sato S, Shiozaki T, Kato M, et al. A new and simple method for evaluating masticatory function using newly developed artificial test food. J Oral Rehabil. 2003;30(1):68-73.

23. Schneider G, Senger B. Coffee beans as a natural test food for the evaluation of the masticatory efficiency. J Oral Rehabil. 2001;28(4):342-8.

24. Schneider G, Senger B. Clinical relevance of a simple fragmentation model to evaluate human masticatory performance. J Oral Rehabil. 2002;29(8):731-6.

25. Shi CS, Guan QY, Guo TW. Masticatory efficiency determined with direct measurement of food particles masticated by subjects with natural dentitions. J Prosthet Dent. 1990;64(6):723-6.

26. Shiau YY, Peng CC, Hsu CW. Evaluation of biting performance with standardized test-foods. J Oral Rehabil. 1999;26(5):447-52.

27. Slagter AP, Olthoff LW, Steen WH, Bosman F. Comminution of food by complete-denture wearers. J Dent Res. 1992;71(2):380-6.

28. Sugimoto K, Hashimoto Y, Fukuike C, Kodama N, Minagi S. Image analysis of food particles can discriminate deficient mastication of mixed foodstuffs simulating daily meal. J Oral Rehabil. 2014;41(3):184-90.

29. Sugimoto K, Iegami CM, Iida S, Naito M, Tamaki R, Minagi S. New image analysis of large food particles can discriminate experimentally suppressed mastication. J Oral Rehabil. 2012;39(6):405-10.

30. van der Bilt A, Abbink JH, Mowlana F, Heath MR. A comparison between data analysis methods concerning particle size distributions obtained by mastication in man. Arch Oral Biol. 1993;38(2):163-7.

31. van der Bilt A, Fontijn-Tekamp FA. Comparison of single and multiple sieve methods for the determination of masticatory performance. Arch Oral Biol. 2004;49(3):193-8.

32. van der Bilt A, van der Glas HW, Mowlana F, Heath MR. A comparison between sieving and optical scanning for the determination of particle size distributions obtained by mastication in man. Arch Oral Biol. 1993;38(2):159-62.

33. Van der Glas H, Al-Ibrahim A, Lyons MF. A STABLE ARTIFICIAL TEST FOOD SUITABLE FOR LABELING TO QUANTIFY SELECTION AND BREAKAGE IN SUBJECTS WITH IMPAIRED CHEWING ABILITY. J Texture Stud. 2012;43(4):287-98.

34. Van der Glas HW, Van der Bilt A, Olthoff LW, Bosman F. Measurement of selection changes and breakage functions during chewing in man. J Dent Res. 1987;66(10):1547-50.

35. Yurkstas A, Manly RS. Value of different test foods in estimating masticatory ability. J Appl Physiol. 1950;3(1):45-53.

36. Yurkstas A, Manly RS. A VOLUMETRIC METHOD FOR MEASURING MASTICATORY PERFORMANCE WITH DIFFERENT TEST FOODS. J Dent Res. 1950;29(5):675-6.
