# Supplementary material for: Systematic review of measurement properties of methods for objectively assessing masticatory performance
Source: Clin Exp Dent Res. 2019 Jan 31;5(1):76–104. doi: 10.1002/cre2.154 (PMC6392827; doi:10.1002/cre2.154)
Supplement: Supplementary file 3 — Data S3 Supporting information [file CRE2-5-76-s003.docx]

^1-7^

1. Fujiyama N, Masuda G, Fukagai T, Ohnuki H, Masuda M, Ohtani T, et al. A new method for the evaluation of masticatory efficiency. The Journal of the Japanese Academy of Maxillofacial Prosthetics. 1979;2:76.

2. Hirano K, Takahashi Y, Hirano S, Hayakawa I, Seki T. A Study on Measurement of Masticatory Ability Using a Color-changeable Chewing Gum with a New Coloring. Nihon Hotetsu Shika Gakkai Zasshi. 2002;46(1):103-9.

3. Imamura T. A new spectrophotometric method for simple determination of masticatory efficiency. J Jpn Prosthodont Soc. 1979;23:603.

4. Kasahara S, Ohtsuka S, Takahashi Y, Komatsu J, Sekido T, Shibasaki Y, et al. New Measurement of Masticatory Performance Using Chewing-Gum Part 1 Measurement and Properties of Test Food. Journal of Japanese Cleft Palate Association. 1989;14(2):205-15.

5. Masuda G, Fujiyama N, Koga I, Fukagai T, Masuda M, Ohtani T, et al. The new method of measuring masticatory performance using spectrophotometer with ATP granules. 1st evaluation of measuring method and materials. J Jpn Stomatol Soc. 1981;30:103-10.

6. Numata O. [A method for evaluation of compressive ability in mastication by chewing gum containing polycarbonate microcapsules]. Kokubyo Gakkai zasshi The Journal of the Stomatological Society, Japan. 1989;56(4):513-27.

7. Tanimoto Y, Watanabe M, Kono R, Hirota C, Takasaki K, Kono K. [Utility of color-changeable chewing gum to evaluate masticatory ability in community-dwelling elderly persons]. [Nihon koshu eisei zasshi] Japanese journal of public health. 2009;56(6):383-90.

8 . Moreschi PE. Desenvolvimento e caracterização de micropartículas "beads" para avaliação da eficiência mastigatória [dissertação]. Ribeirão Preto: Faculdade de Ciências Farmacêuticas, Universidade de São Paulo; 2006.

9. Mazzetto MO, Hotta TH, Petenusci SO, Mestriner-Junior W, Yamasaki MK, et al. Eficiência mastigatória: Análise de correlação de dois testes. Revista Gaúcha de Odontologia. 2010; 5: 319-322.
