# Supplementary material for: Systematic review of measurement properties of methods for objectively assessing masticatory performance
Source: Clin Exp Dent Res. 2019 Jan 31;5(1):76–104. doi: 10.1002/cre2.154 (PMC6392827; doi:10.1002/cre2.154)
Supplement: Supplementary file 4 — Table S1 Supporting information [file CRE2-5-76-s004.docx]

|  | **Validity (criterion validity)** | | | **Validity (hypothesis testing)** | | | **Responsiveness** | | | **Reliability** | | | **Measurement error** | | |
| --- | --- | --- | --- | --- | --- | --- | --- | --- | --- | --- | --- | --- | --- | --- | --- |
| **Study, first author** | **Reported** | **COSMIN score** | **Quality criteria** | **Reported** | **COSMIN score** | **Quality criteria** | **Reported** | **COSMIN score** | **Quality criteria** | **Reported** | **COSMIN score** | **Quality criteria** | **Reported** | **COSMIN score** | **Quality criteria** |
| Silva 2018 |  |  |  |  |  |  |  |  |  | Visual assessment:  Inter-rater, overall weighted kappa 95%CI 0,81(0,78-0,84)  Intra-rater, overall weighted kappa 95%CI 0,83(0,76-0,89). | Good | Positive | Colorimetry VOH measuurements, mean difference 0,0005, LoA 95%CI 0,0034-(-0,0024) | Good | Indeterminate |
| Khoury-Ribas 2017 |  |  |  | Correlation between using Optozeta and Optosil as test food. P<0,001. Median particle size: 0,807 Preferred chewing side (1 cycle): 0,823 Preferred chewing side (all cycles): 0,884 Chewing cycle duration: 0,874 | Poor | Positive |  |  |  | Test-retest  Median particle size ICC (CI 95%) Optosil: 0,62 (0,06-0,89) Optozeta: 0,67 (0,10-0,91)  Preferred chewing side ICC (CI 95%) Optosil: First cycle 0,64 (0,05-0,90), all cycles 0,79 (0,38-0,94) Optozeta: First cycle 0,92 (0,73-0,98), all cycles 0,90 (0,64-0,97)  Chewing cycle duration ICC (CI 95%) Optosil: 0,63 (0,10-0,89) Optozeta: 0,76 (0,29-0,93) | Poor | Negative |  |  |  |
| Wada 2017 |  |  |  | Preparation of food with suitable food texture for swallowing and association with a* value of color changing chewing gum (crude odds ratios, 95%CI): Boiled rice 1,18 (1,07-1,36) p<0,001, ginger-fried pork loin 1,15 (1,01-1,45) p<0,026. Boiled fish-paste 1,17(1,06-1,35) p<0,001, rice cracker 1,50(1,17-2,81) P<0,001. No association with white sliced bread.   Cut off values a*: Boiled rice 27,8 sensitivity 0,76 specificity 1,00, pork loin 28,7 sensitivity 0,86, specificity 0,65. Fish paste 21,2 sensitivity 0,94 specificity 1,00, rice cracker sensitivity 0,88 specificity 0,75. | Fair | Positive |  |  |  |  |  |  |  |  |  |
| Goto 2016 |  |  |  | Significant correlation between OIV after 10s chewing duration and gummy jelly-related masticatory efficiency test value, r=0,611 p<0,05. No other values statistically significant. | Poor | Positive |  |  |  |  |  |  | CoV: Odour intensity masticatory value lower than for gummy jelly test food (golden standard). Only diagram presented, no data. | Poor | Indeterminate |
| Sanchez-Ayala 2016 | Measurment means of FB compared with SC and sieve method. FB mean (absorbance %)= 28,86 ±16,85. SC mean(X_50_%)=60,62 ±12,37.   Average of absolute difference 31,75 ±17,12 p<0,001. | Good | Negative |  |  |  |  |  |  | Intra-rater Fuchsin beads ICC=0,41-0,83 Sieve method ICC=0,86-0,97 Inter-rater Fuchsin beads ICC=0,56-0,89 Sieve method ICC=0,83-0,96 | Good | Indeterminate(Fuchsin beads) Positive (Sieve method) |  |  |  |
| Vaccaro 2016 |  |  |  | Correlation VhH and CS r=0,792, p<0,0001. | Excellent | Positive |  |  |  |  |  |  |  |  |  |
| Eberhard et al (2015) | Correlation of PD between scanning & sieve method.  CS=15 r=0.995 p<0.01 CS=40 r=0.971 p<0.01  Bland-Altman plot CS=15 All X_50_ values w/in ±10% range. 86% of values w/in ±5% range.  CS=40, scanning tend to overestimate particles size.  LoA not presented. | Poor | Positive |  |  |  |  |  |  |  |  |  |  |  |  |
| Schimmel 2015 |  |  |  | VOH decrease with increasing #CS. CS could be predicted with VOH for all 3 gums, r^2^= 0,8909, 0,8938 and 0,8909, p<0,0001 for all. | Fair | Positive |  |  |  | Subjective assessment bolus/wafer (Gum1 not used): CS=5= Gum2 intra- k=0,7479 inter- k=0,5935 Gum3 intra k=0,6512 inter- k=0,4186 CS=10 Gum2 intra- k=0,5902 inter- k=0,7521 Gum3 intra- k=0,3496 inter- k=0,7500 CS=20 Gum2 intra- k=0,6250 inter- k=0,8750, Gum3 intra- k=-0,1364 inter- k=0,6429 CS=30 Gum2 intra- k=0,8571 inter- k=0,8571, Gum3 intra- k=0,4595 inter- k=0,7727  Note best agreement with Gum2 at CS=30  Cumulative kappa over all CS:  Gum2 intra-k=0,9018, inter- k= 0,9123., Gum3 intra-. k=0,8206, inter- k=0,9430 | Fair | Positive  for CS=20/30 | Colorimetry VOH measurements. Gum2 intra-rater mean difference 0,000, LoA 95% CI: -0,0001-0,0001 Gum3 mean difference 0,002, LoA 95% CI: 0,000-0,003 Inter-rater Gum2 mean difference 0,000, LoA 95% CI -0,0001-0,0001. Gum3 mean difference 0,006, LoA 95% CI 0,001-0,010 | Fair | Indeterminate |
| Endo 2014 |  |  |  |  |  |  |  |  |  | inter-, intra-rater  Kappa value. Inter-rater  CS= 10: Bolus shape k=NR. Colormixing bolus k=0,52. Wafer colormixing k=0,23. CS=20:Bolus shape k=0,07. Bolus colormixing k= 0,50. Wafer k=0,45 CS=30: Bolus shape k=0,12. Colormixing bolus k=0,42. Wafer colormixing k=0,59. CS=50: Bolus shape k=0,10. Colormixing bolus k=0,51. Wafer colormixing k=0,46.  Intra-rater CS=10: Bolus shape k=?. Colormixing bolus k=0,27. Wafer colormixing k=0,28. CS=20:Bolus shape k=0,44. Bolus colormixing k= 0,65. Wafer k=0,54 CS=30: Bolus shape k=0,49. Colormixing bolus k=0,90. Wafer colormixing k=0,57. CS=50: Bolus shape k=0,52. Colormixing bolus k=0,78. Wafer colormixing k=0,47.  Optical scanning Inter-rater No statistical difference (systematic error) between tests above CS=20 p=0,115-0.998  intra-rater No statistical difference (systematic error) between tests above CS=20 p=0,769-0,864. | Subjective assessment Fair  Optical scanning Poor | Negative  Indeterminate |  |  |  |
| Hama 2014 |  |  |  | ΔE increased with #CS.  Significant difference (p<0,001) in ΔE between  dentate and edentulous groups. Median value (25th-75th percentile): Dentate 126,1 (101,5-142,1)  Edentulous 71,7 (53,2-84,8). | Poor | Positive |  |  |  | Test-retest: CS=20, ICC=0,46 CS=40, ICC=0,66 CS=60, ICC=00,79 CS=80, ICC=0,83 CS=100, ICC=0,82 CS=120, ICC=0,83 CS=160, ICC=0,74 CS=200, OCC=0,64  No CI presented | Poor | Positive  for CS=80-120 |  |  |  |
| Hama 2014 |  |  |  | Correlation of color change assessed with color scale and colorimeter. Dentist: r=0,96 p<0,001 Students: r=0,97 p<0,001 Elderly: r=0,96 p<0,001 | Fair | Positive |  |  |  | Intra-rater  Dentist group: ICC  0,96, 0,97, 0,93, 0,96, 0,95, 0,92.  Student group: ICC 0,98, 0,95, 0,96, 0,98, 0,96, 0,98  Elderly: ICC 0,96, 0,94, 0,98, 0,96, 0,97, 0,94  Inter-rater  Dentist: 0,93, Students: 0,96, Elderly: 0,92. All groups: 0,94  No CI presented. | Fair | Positive |  |  |  |
| Halazonetis 2013 |  |  |  | Correlation median SDHue and number of CS. r^2^=0,94, p<0,01) | Fair | Positive |  |  |  |  |  |  | inter-, intra-rater  SDHue  Intra-rater: Average of the differences 0,0004. LoA 95% CI:  -0,008, 0,009  Intra-rater: Average of the differences 0,003. LoA 95% CI:  -0,021, 0,015 | Fair | Indeterminate |
| Nokubi 2013 |  |  |  | Significant correlation between visual scoring scale and glucose concentration method, p<0,001 in all three measurments: 1st r=0,915-0,978, 2nd 0,911-0,981, 3rd r=0,912-0,975. | Fair | Positive |  |  |  | Inter-rater ICC: First test, ICC=0,946, second test, ICC=0,947, third test, ICC=0,946. No CI presented.   Intra-rater ICC: ICC range 0,860-0,987 | Good | Positive |  |  |  |
| Sanchez-Ayala 2013 |  |  |  |  |  |  |  |  |  | Inter-, intra-rater  All lot of data presented in article.  We refer to article for exact data.  Intra-rater: ICC=≥0,92 Inter-rater: ICC≥0,93  CI presented for each ICC data | Good | Positive | Absolute mean of absolute difference <1mm for multiple sieve method.  Similar results for single-/multiple-sieve method | Fair | indeterminate |
| Weijenberg 2013 |  |  |  | Correlation between two colored gum test and two colored wax test, r=0,429. | Poor | Negative |  |  |  | Test-retest  ICC=0,714, no CI presented. | Poor | Positive |  |  |  |
| Eberhard 2012 | Correlation of PD between scanning and sieve method.  r=0,919 p<0,01  Bland-Altman plot Average of differences X_50_mm 0,05 LoA 95% CI 0,10, -0,05 | Poor | Positive |  |  |  |  |  |  |  |  |  |  |  |  |
| van det Bildt 2012 |  |  |  | Correlation of visual assessment of MAI compared to digital analysis. r=0,95 p<0,001. | Good | Positive |  |  |  | Test-retest k=0,7-0,78 Inter-rater 0,55<k<0,65 | Fair | Positive |  |  |  |
| Abe 2010 |  |  |  | BFI rice: CS=10 63,0 ±6,6%, CS=15 69,5 ±6,6%, CS=20 74,9 ±5,6%, CS=30 85,8 ±2,9%. Significant correlation with CS r=0,84 p<0,01.  BFI uirou: CS=10 64,5 ±6,3%, CS=15 72,3 ±5,5%, CS=20 79,6 ±4,8%, CS=30 89,4 ±4,1%. Significant correlation with CS r=0,89 p<0,01.  BFI rice under "chewing normally"/"chewing well": 72,4 ±7,8% / 89,5 ±6,7%.  BFI uirou under "chewing normally"/"chewing well": 78,7 ±9,9% / 92,6 ±7,8% | Poor | Positive |  |  |  |  |  |  |  |  |  |
| Ikebe 2010 |  |  |  | MP of groups B and C were 81% and 49% of that of group A. | Good | Positive |  |  |  |  |  |  |  |  |  |
| Kamiyama 2010 | Correlation between a* and mean color score, r=0,979 | Fair | Positive |  |  |  |  |  |  | Inter-rater: Dentists: ICC=0,91, students: ICC=0,89, elderly: ICC=0,86.  Intra rater: Dentist: Average ICC 0,94 (0,91, 0,92, 0,93, 0,95, 0,97, 0,97) Students ICC 0,89 (0,8, 0,85, 0,9, 0,91, 0,93, 0,96) Elderly ICC 0,88 (0,81, 0,86, 0,87, 0,89, 0,91, 0,94)  No CI presented. | Fair | Positive |  |  |  |
| van der Bildt 2010 |  |  |  | Correlation between gum test and comminution test. CS=10 young r=0,20-0,31, elderly r=0,50, all r=0,56 CS=20 young r=0,10-0,17, elderly r=0,67, all r=0,64 | Fair | Negative four young group Positive for elderly |  |  |  |  |  |  |  |  |  |
| Woda 2010 |  |  |  | 62% of aged denture wearers produced food bolus with median particle size above MNI, 4,0mm, compared with aged dentate of 2%. | Poor | Positive |  |  |  |  |  |  |  |  |  |
| Speksnijder 2009 |  |  |  | Significant correlation 0,52-0,66 p<0,000  between wax mixing test and comminution test. Statistical difference between denture groups when using wax cube and CS=15 or 20, p<0,033 and 0,006 respectively. No statistical difference between denture groups in comminution test. | Good | Positive |  |  |  |  |  |  |  |  |  |
| Sugiura 2009 |  |  |  | Correlation MAI and comminution test: Dentate group, r=-0,56 p<0,001, edentate -0,7 p<0,001. Correlation MAI and gummy jelly test: Dentate group, r=-0,12 p<0,53, edentate r= 0,21 p<0,19. | Fair | Positive |  |  |  |  |  |  | MAI mean difference,95% CI= 0,07 (-0,10-0,24), LoA=-0,41-0,55 | Good | Indeterminate |
| Fauzza 2008 |  |  |  |  |  |  | Particle weight (median %):  Mesh 2.0mm  CS=10 old denture 98,60%  new denture 97,98%.  CS=20 old denture 95,37%, new denture 93,07%.  Mesh 1,7mm:  CS=10 old denture 0,54%, new denture 0,55%  CS=20 old denture 1,37%, new denture 1,91%.  Mesh 1,4mm:  CS=10 old denture 0,28%, new denture 0,23%.  CS=20 old denture 1,02%, new denture 1,47%.  Mesh 1,0mm:  CS=10 old denture 0,33%, new denture 0,44%.  CS=20 old denture 0,88%, new denture 1,44%.  Filter paper: CS=10 old denture 0,42%, new denture 0,46%. CS=20 old dentures 1,56%, new dentures 1,95%.  No statistical differences between old and new denture. | Poor | Negative |  |  |  |  |  |  |
| Felicio 2008 |  |  |  | Correlation with EMG activity, r=0,76 p<0,01 | Poor | Positive |  |  |  | Split-half reliability test.  Relation of fuchsin beads in relation to EMG activity r=0,86, p<0,01 | Poor | Positive |  |  |  |
| Lujan-Climent 2008 |  |  |  |  |  |  |  |  |  | ICC=0,97(0,88-0,99) 95% CI | Poor | Positive | Smallest detactable difference: median particle size=4mm | Poor | Indeterminate |
| Ishikawa 2007 |  |  |  |  |  |  | Mean difference between old and new denture, Δa*, 1,14 ±6,41 p>0,05. No correlation between gum and comminution test, r=-0,027 p>0,05. | Poor | Negative |  |  |  |  |  |  |
| Schimmel 2007 |  |  |  | UF decreased significantly  with increased #CS, 0,0001<p<0,0004 | Poor | Indeterminate |  |  |  | Inter-rater CS=5: Bolus k=0,30, Wafer k=- 0,21 CS=10: Bolus k=0,50, Wafer k=0,13 CS=20: Bolus k=0,51, Wafer k=- 0,52 CS=30: Bolus k=0,66, Wafer k=- 0,47 Intra-rater (examiner1/examiner/2) CS=5: Bolus k=0,63/0,51, Wafer k=0,88/0,69 CS=10: Bolus k=0,40/0,20, Wafer k=063/? CS=20: Bolus k=0,62/0,41, Wafer k=0,78/0,68 CS=30: Bolus k=0,52/0,20, Wafer k=0,60/0,67 | Poor | Negative |  |  |  |
| Escudeiro Santos 2006 |  |  |  |  |  |  |  |  |  | Intra-individual analysis No statistical significance difference,p<0,005, between the 9 capsules chewed in three evaluation tests. Inter-individual analysis | Poor | Indeterminate |  |  |  |
| Ikebe 2006 |  |  |  | Linear regression showed correlation between glucose concentration and surface area (mm^2^) of comminuted jelly, r=0,993 p<0,001. | Poor | Positive |  |  |  |  |  |  |  |  |  |
| Kobayashi 2006 | Correlation gummy jelly glucose extraction and masticatory value (sieve method) for CS=20 or 30 r=0,799-0,850 p<0,01-0,05 | Poor | Positive |  |  |  |  |  |  |  |  |  |  |  |  |
| Shiga 2006 |  |  |  | Correlation  Between blood glucose meter and spectrophotometer  r=0,994 p<0,01 | Poor | Positive |  |  |  |  |  |  |  |  |  |
| Azakawa et al. 2005 |  |  |  |  |  |  | Difference of MAI between old/new denture (mean ±SD).  MAI old dentures: -0,11 ±1,13 MAI new dentures: 0,70 ±0,68 p<0,001 | Fair | Indeterminate |  |  |  |  |  |  |
| O´Hara 2003 |  |  |  | Significant correalation between CS and particle number/weight of test food for each sieve p<0,01-0,05. Higher correalation to CS than peanuts.  A lot of data presented in article. Please see reference for complete data set. | Poor | Positive |  |  |  | Intra-rater: Significant positive correlation between morning, noon, after-noon. r=0,68-0,89, p=<0,05-<0,01. No significant difference between sieves 2,0, 1,7, 1,4, 1,18, 1,0, 0,85mm.  Test-retest: Significant positive correlation between 3 different days. r=0,65-0,90, p=<0,05-<0,01. No significant difference between sieves 2,0, 1,7, 1,4, 1,18, 1,0, 0,85mm | Fair | Negative |  |  |  |
| Sato 2003 |  |  |  | Correlation between MAI and CAI. Group A: r=0,66 p<0,001 Group B: r=0,72 p<0,001 Group C: r=0,56 p<0,001 | Fair | Positive |  |  |  | Inter-rater: ICC=0,94 Intra-rater: ICC=0,98 Test-retest: ICC=0,89 Measureing consistency: ICC=0,99  No CI presented. | Poor | Positive |  |  |  |
| Prinz et al. 1999 |  |  |  | Correlation of image processing measures and CS after CS=10.  Low contrast r=0.5 p<0.001  High contrast r=-0.2 p<0.05  Polarity r=.0.13 p=n.s  Fragmentation r=-0.24 p<0.05  Blending r=0.07 p=n.s  Spatial frequency r=0.14 p<0.05  Nearest neighbor analysis r=0.21 p<0.05 | Poor | Negative |  |  |  | No significant difference between raters (p>0.05.  Pair-wise t-test:  Bolus gum, error=2.65, SD=0.92  Flattened gum,  Error=1.93, SD=0.72,p<0.05  Digital image processing, Error=3,62, SD=2,53. | Poor | Indeterminate |  |  |  |
| Hayakawa 1998 |  |  |  | Test 1: Correlation color change, a*, and CS, r= 0,93 p<0,01. Test 2: % of correct answers by examiners using color scale. Examiner A: CS=5 83%, CS= 15 83%, CS=30 67%, CS=45 67%. Examiner B: CS=5 83%, CS=15 67%, CS=30 83%, CS=45 83/%. Examiner C: CS=5 83%, CS=15 67%, CS=30 67%, CS=45 67% Examiner D: CS=5 83%, CS=15 67%, CS= 30 67%, CS=45 83%. | Poor | Positve |  |  |  |  |  |  |  |  |  |
| Huggare 1997 |  |  |  | Correlation of absorbed color and reciprocal of the particle diameter (1/D), of two different colorbinders (1% Praestol and 2%Praestol), 0,9974 and 0,9975 respectively. | Poor | Positive |  |  |  | Test re-test  Visual assessment:  Inter-rater, overall weighted kappa 95%CI 0,81(0,78-0,84)  Intra-rater, overall weighted kappa 95%CI 0,83(0,76-0,89).  No significant difference between tests of the 4 individuals.  Mean difference and SD:  1,130 ±0,038, 1,165 ±0,0206, 0,757 ±0,0334, 0,507 ±0,040 | Poor | Indeterminate |  |  |  |
| Matsui 1996 |  |  |  | Relationship a* and CS.  As CS increased so did a*. Only diagram presented, no data.  Statistical difference between dentate/edentate groups p<0,001.  Average a*, dentate 28,76 ±1,76 95%CI, edentate 10,05 ±2,58 95%CI. | Poor | Positve |  |  |  |  |  |  | CoV:  Dentate group mean 5,15 range 1,87-7,9% Edentate group mean 9,75% range 1,5-17,9% | Poor | indeterminate |
| Mowlana 1994 |  |  |  | Median particle size mm (X_50_), b=broadness variable CS=1 Scan X_50_= 4,94±0,36 b=2,11 ±0,12 Sieve X_50_=5,71 ±1,08 b=1,73 ±0,09. CS=4 Scan X_50_=2,59 ±0,31 b=1,37 ±0,02  Sieve X_50_=2,17 ±0,27 b=1,39 ±0,05  CS=8 Scan X_50_=1,44 ±0,20 b=1,22 ±0,04 Sieve X50=1,22 ±0,15 b=1,49 ±0,07 CS=16 Scan X_50_=0,85 ±0,09 b=1,35 ±0,05 Sieve X50=0,80 ±0,07 b= 1,68 ±0,07 CS=32 Scan X_50_=0,72 ±0,04 b=1,46 ±0,13 Sieve X_50_=0,69 ±0,05 b=1,79 ±0,10  Median partical size significantly larger with optical scanning compared to sieve at CS=4,8,16 | Poor | Indeterminate |  |  |  |  |  |  |  |  |  |
| Slagter 1993 |  |  |  | Correlation between Optosil and Optocal: Dentition group CS=20, 40, 60, 80, r=0,68-0,88 Complete denture group CS=20, 40, 60, 80, r=0,56-0,98 | Poor | Positive |  |  |  |  |  |  |  |  |  |
| Mahmood 1992 |  |  |  |  |  |  | Difference of MP before and after new denture not statistical significant. Mean area of particle (mm^2^): Before 9,9 ±7,1. After 7,8 ±4,7. | Poor | Negative | No significant difference between tests (mean particle area mm^2^). Subject A: 1st measurement 1,99 ±7,03, 2nd 2,02 ± 7,19 p<0,11. Subject B: 1st 2,02 ±2,43, 2nd 2,27±3,46 p<0,88. | Poor | Indeterminate |  |  |  |
| Gunne 1985 |  |  |  | No correlation between gelatin test and sieve test (data not presented).  MP described as % of dentate group performance, then partial dentures performed 80-83% and complete dentures 61-69%. | Poor | Indeterminate |  |  |  | Variance components: No significant systematic difference between examiners. | Poor | Indeterminate |  |  |  |
| Kapur 1964 |  |  |  |  |  |  |  |  |  | Test1: ICC=0,95. No CI presented. Test2: ICC=0,96. No CI presented. | Fair | Positive |  |  |  |
| **CS= chewing cycles, #CS=number of chewing cycles, MP= Masticatory performance, NR= Not reported, LoA=Limits of Agreement, UF=Unmixed fraction of pixels, SDHue=Standard deviation of hue, MAI=Mixing Ability Index, ΔE=Color change, L*=lightness of color, a*=color between red/green,. b*= color between yellow/blue, VOH=Variance of hue, SDirb=Standard deviation of intensity of distrubution red and blue color, ΔA=difference in light absorption, X_50_= Theoretical sieve aperture value were 50% of particle volumes can pass, median particle size, b=Broadness of, distribution of particles sizes, PD=particle size distribution, ST=swallowing threshold, AU=Absorbence units, GS=Gold standard, FB=Fuchsine beads, SC=Silicone cubes, BFI=Bolus formation index A=total area of bolus (in pixels).W=white area of bolus BFI=(A-W)/A, OIV= Odour intensity value (mean and SD), SDid= Standard deviation of intensity distribution, VhH= Variance of histogram of Hue,MRI=Masticator normative indicator, EMG=Electromyography analysis, wgt.=weight, n.s=not significant, k=kappa value, ICC=Intraclass correlation coefficient**  **, CI= Confidence interval, LoA=Limits of Agreement, CoV=Coefficient of variation.** | | | | | | | | | | | | | | | |
